# The Shapes of Sulfonamides: A Rotational Spectroscopy Study

**DOI:** 10.3390/molecules27092820

**Published:** 2022-04-28

**Authors:** Annalisa Vigorito, Camilla Calabrese, Assimo Maris, Donatella Loru, Isabel Peña, M. Eugenia Sanz, Sonia Melandri

**Affiliations:** 1 Dipartimento di Chimica “G. Ciamician” dell’Università, via Selmi 2, I-40126 Bologna, Italy; vigogiff@gmail.com (A.V.); calabrese.cami@gmail.com (C.C.); assimo.maris@unibo.it (A.M.); 2 Department of Chemistry, King’s College London, Britannia House, 7 Trinity Street, London SE1 1DB, UK; donatella.loru@desy.de (D.L.); mipencal@uva.es (I.P.); maria.sanz@kcl.ac.uk (M.E.S.)

**Keywords:** rotational spectroscopy, supersonic expansion, molecular structure, ab initio calculations, sulfonamides, sulfanilamide

## Abstract

Benzenesulfonamides are a class of molecules of extreme interest in the biochemical field because many of them are active against a variety of diseases. In this work, the pharmacophoric group benzensulfonamide, its derivatives *para-*toluensulfonamide and *ortho-*toluensulfonamide, and the bioactive molecule sulfanilamide, were investigated using rotational spectroscopy to determine their conformations and the influence of different substituents on their structures. For all species, the hyperfine structure due to the ^14^N atom was analyzed, and this provided crucial information for the unambiguous identification of the observed conformation of all molecules. In addition, for *ortho*-toluensulfonamide, the vibration–rotation hyperfine structure related to the methyl torsion was analyzed, and the methyl group rotation barrier was determined. For benzensulfonamide, partial *r*_S_ and *r*_0_ structures were established from the experimental rotational constants of the parent and two deuterated isotopic species. In all compounds except *ortho*-toluensulfonamide, the amino group of the sulfonamide group lies perpendicular to the benzene plane with the aminic hydrogens eclipsing the oxygen atoms. In *ortho*-toluensulfonamide, where weak attractive interactions occur between the nitrogen lone pair and the methyl hydrogen atoms, the amino group lies in a *gauche* orientation, retaining the eclipsed configuration with respect to the SO_2_ frame. A comparison of the geometrical arrangements found in the PDB database allowed us to understand that the bioactive conformations are different from those found in isolated conditions. The conformations within the receptor are reached with an energy cost, which is balanced by the interactions established in the receptor.

## 1. Introduction

In biological chemistry, the structural characterization of a ligand within its biological target is of extreme importance. For instance, in ligand-based drug design, the ligand’s bioactive conformation is used to infer the necessary characteristics that a molecule must possess to bind to the target. These features allow us to define a pharmacophore model that may be used to design new molecular entities to interact with the target [1]. However, if on one side the role of the ligand’s bioactive conformation is well recognized, then on the other side, the free ligand conformation is often considered to be of secondary importance. Nevertheless, in a recent study [2] it was pointed out that, for a drug ligand, the binding to the target and the strength of the effect are related to its ability to adopt the right shape to fit into the target binding site. If a molecule already possesses a good binding shape (i.e., a bioactive conformation), it is likely to bind strongly to its target and be a good drug, while the opposite is true for molecules which need to change considerably in order to fit. Therefore, knowledge of the 3D-shape of the free ligand is extremely valuable in guiding the design of the best drug molecules. The ligand 3D-shape should be determined under isolated conditions in the gas phase, where the molecules are essentially free of intermolecular interactions, so that their intramolecular interactions can be clearly modelled and the energy costs associated with different conformations in the receptor pocket can be estimated.

Compounds belonging to the sulfonamide class, in particular those containing the benzosulfonamide group (Ph-SO_2_NH_2_), are of extreme interest in the biological field because many of them are active against a variety of diseases. Sulfanilamide was the first clinically useful antibacterial drug [3]. Today, derivatives such as sulfamethoxazole are largely used for treating urinary tract infections and bronchitis and are effective against both Gram-negative and Gram-positive bacteria. The microbial activity of sulfonamides is exerted through the deactivation of the dihydropteroate synthase enzyme [3]. In the receptor binding site, they act as analogues of the natural substrate *para*-aminobenzoic acid, producing folate and resulting in bacterial death. The X-ray crystal structure is available and catalogued in the RCSB Protein Data Bank with the code 3TZF.

In addition, relatively recent investigations have found that aryl-sulfonamides are extremely potent inhibitors of the metalloenzyme family, carbonic anhydrases [4]. These enzymes catalyze the reversible hydration of carbon dioxide to the bicarbonate ion and proton, a crucial equilibrium involved in many physiological processes such as respiration, pH balance and ion transport, and their inhibition is useful for the treatment of a multitude of diseases [4,5,6]. Several aryl-sulfonamides acting as antagonist ligands of these enzymes have been FDA-approved as anti-glaucoma, anti-inflammatory [7], anti-tumor [8] and anti-viral HIV drugs, and as medicines for the treatment of Alzheimer’s disease. X-ray crystal structures available for aryl-sulfonamide compounds in isoenzyme carbonic anhydrases are catalogued in the PDB with the codes 4COQ, 2HL4, 1JSV, 4JSA and 3K34.

**Figure 1:**
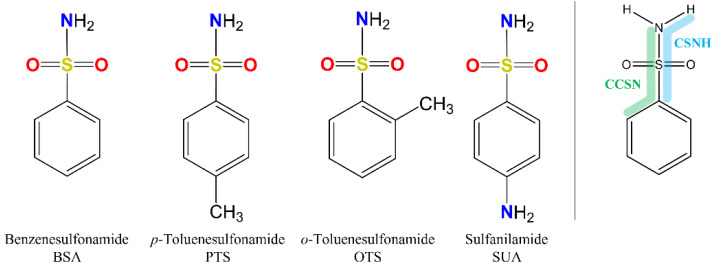
Chemical structures of BSA, PTS, OTS and SUA, and schematic indicating their characterizing dihedral angles.

Because of the profound biological interest in the sulfonamide class, we addressed the structural characterization, in the gas phase, of their main pharmacophore group, benzensulfonamide (BSA), its methyl derivatives *ortho*-toluensulfonamide (OTS) and *para*-toluensulfonamide (PTS), and the drug sulfanilamide (SUA) (see Figure 1). Their structures were investigated using rotational spectroscopy, a spectroscopic technique which allows determination of the conformational preferences of the target compounds with extraordinary accuracy. Due to the presence of the ^14^N quadrupolar nucleus, these spectra may also exhibit hyperfine structures, leading to the determination of the ^14^N quadrupole coupling constants. The latter, in addition to the rotational constants, constitute a tool for the unambiguous identification of different conformers.

Previous studies using rotational spectroscopy show that flexible chains, composed of different atoms such as C, O, N or S and attached to an aromatic ring, give rise to several configurations. The structural variability is also observed in systems with short side chains. In the prototype compound of this series, ethylbenzene [9], the ethyl group is perpendicular to the ring plane, while in anisole [10] the side chain lies in the plane of the aromatic ring. In benzylamine [11], two conformations have been observed: in one of them, the amino group is in the *gauche* position with respect to the aromatic plane, with the nitrogen lone pair pointing toward one of the aromatic hydrogen atoms, while in the second conformer the amino group lies perpendicular to the aromatic plane with the amino hydrogens pointing toward the π-cloud. In the first conformer, the inversion motion of the −CH_2_NH_2_ group above and below the phenyl plane, was also observed and modelled. In the case of the benzyl alcohol [12], four equivalent conformers in which the alcohol group is in *gauche* orientation with respect to the aromatic plane were determined.

Regarding benzene compounds with a side chain containing sulphur, the conformational equilibrium of methyl phenyl sulfoxide [13] and methyl-4-nitrophenyl sulfoxide [14] were experimentally investigated in the gas phase using microwave spectroscopy, and in isotropic and nematic liquid crystal solutions using NMR spectroscopy. These studies have shown that both methyl phenyl sulfoxide and methyl-4-nitrophenyl sulfoxide favor a conformation stabilized by intramolecular interactions (in particular, optimization between steric repulsion and conjugation of π systems) although significant solvent effects have been also detected. Among the benzenesulfonyl compounds, only benzene sulfonyl chloride has been investigated by rotational spectroscopy [15]. In this case the Cl atom lies perpendicular to the benzene plane.

On the subject of sulfa-drugs, a combined study of Gas Electron Diffraction (GED) and quantum chemical calculations has been reported for BSA [16], PTS and OTS [17]; while a combined matrix isolation Fourier Transform Infrared Spectroscopy (FTIR) and theoretical DFT study [18], as well as conformer-specific UV and IR spectroscopy studies [19], have been conducted on SUA. Overall, the following theoretical results were found: two different conformations exist for all of the molecular species in which the S–N bond is perpendicular (or almost perpendicular) to the benzene ring and the –NH_2_ group staggering or eclipsing the –SO_2_ group. When a substituent is present, other conformations arise from the staggered and eclipsed ones originating from the different orientation of the substituents. These studies are quite extensive, but not conclusive, in regard to the observed structures. This is because the structural information coming from the GED experiment is only an average over all of the vibrational states that are populated at the temperature of the measurement; while IR spectroscopy measures the absorption rate of any wavelength within the beam as a whole, and the resolution usually does not allow for a direct distinction between molecular species which differ only by the relative orientation of the functional groups. Hence, these two techniques are not as accurate as rotational spectroscopy, which can provide precise structural and conformational data because it probes rotational transitions at the lowest vibrational ground state, and its much higher resolution allows the observation of dozens of unblended lines for each conformation.

In this paper, we report a rotational spectroscopy study, supported by quantum chemical calculations, of BSA, PTS, OTS and SUA. To the best of our knowledge this study is the first rotational spectroscopy study of these molecules. Thus, besides spectral characterization, which could be useful for their identification, the study unambiguously reveals the conformational preferences of each compound in isolated conditions, allowing us to define the influence of substitution by different groups in different positions.

## 2. Results and Discussion

### 2.1. Computational Results

For the four compounds BSA, PTS, OTS and SUA, different rotamers were generated by the rotation of the sulfonamide and amino groups, the orientations of which are defined by two dihedral angles: ∠CCSN and ∠CSNH, respectively (see Figure 1). Rotation of the methyl group in PTS and OTS is not relevant to describe different rotamers, because it gives rise to three equivalent minima. However, this motion must be taken into consideration because it could generate splitting of the rotational transitions due to the coupling of this internal motion with the overall rotation. For the possible rotamers, full geometry optimizations and subsequent harmonic frequency calculations to characterize the stationary points were run at the B3LYP and MP2/6-311++G** level of theory, using the Gaussian09 programs package [20].

The calculations indicated that the conformational behavior of BSA, PTS and SUA is very similar. For all compounds, two sets of conformers with a symmetry plane perpendicular to the aromatic plane and containing the C−S bond (∠CCSN = 90°) could be identified. They differ in the position of the amino group hydrogen atoms, which can be oriented so as to either eclipse or stagger the oxygen atoms of the –SO_2_ group, and are labelled as 1 and 2, respectively. For the substituted molecules, PTS, OTS and SUA, two additional stable conformations which differ by the orientation of either the methyl group (PTS), the sulfonamide group (OTS), or the amino group (SUA) could be found. They were labelled by the letters *a* and *b*, respectively. The calculated structures and their relative electronic energies (∆*E*), relative zero-point corrected energies (∆*E*_0_) and total dipole moment values are shown in Table 1, while the corresponding spectroscopic parameters are reported in the sections below for each different compound.

By examining the calculated relative energies, we determined that the eclipsed conformers were predicted by both theoretical methods to be the most stable ones. For BSA, the equilibrium energy difference ranges from 2.74 (B3LYP) to 1.24 (MP2) kJ/mol and increases to 3.56 (B3LYP) or 5.00 (MP2) kJ/mol if zero-point energy corrections are taken into account. Similar results are found for the *para-*substituted compounds PTS and SUA. For PTS, four conformers arise both from the eclipsed (PTS1) or staggered (PTS2) orientation of the sulfonyl amino group, and from the methyl group orientation (*a* or *b* conformers). The effect of the different methyl orientations is very small. Considering PTS1a and PTS1b, it varies from 0.55 (B3LYP) to 0.03 (MP2) kJ/mol and is changed to 0.32 (B3LYP) or 0.09 (MP2) kJ/mol if the zero-point energy corrections are considered. In SUA, the *a* and *b* series of conformers arise from the different orientation of the *para*-amino hydrogen atoms, which can be located on the opposite (*a* conformers) or the same (*b* conformers) side of the ring with respect to the sulfonamide group. Likewise in this case, the energy differences between the *a* and *b* conformers are quite small. Considering SUA1a and SUA1b, the relative energies of SUA1b vary from 0.29 (B3LYP) to 0.35 (MP2) kJ/mol and are changed to 0.12 (B3LYP) or −0.28 (MP2) kJ/mol when zero-point energy corrections are included. In this case, the B3LYP values indicate that SUA1a is the global minimum, but the MP2 zero-point corrected values predict SUA1b to be the lowest energy conformation on the potential energy surface.

For OTS, four conformers were also predicted: they are again originated by the amino group, which can be oriented to either eclipse (OTS1) or stagger (OTS2) the oxygen atoms of the −SO_2_ group, and by the same amino group being oriented in either a *gauche* (∠CCSN = 60°; OTS *a* conformers) or planar (∠CCSN = 0°, OTS *b* conformers) position with respect to the benzene plane. Among these conformers, the eclipsed conformations (OTS1a and OTS1b) are lower than the staggered ones (OTS2a and OTS2b), as was found for BSA, PTS and SUA, although the relative energies are higher in this case. Considering the pair OTS1a and OTS1b for example, the relative energies vary from 6.80 (B3LYP) to 5.46 (MP2) kJ/mol and are changed to 6.96 (B3LYP) or 6.70 (MP2) kJ/mol if zero-point energy corrections are taken into account. This energy difference between the staggered and eclipsed conformers is similar to that between the *a* conformers, in which the amino group lies in the *gauche* position, and the *b* ones, in which the amino nitrogen is on the plane. Considering, for example, OTS1a and OTS1b, the relative energies vary from 5.14 (B3LYP) to 5.42 (MP2) kJ/mol and are changed to 4.39 (B3LYP) or 5.80 (MP2) kJ/mol if zero-point energy corrections are included. This energy scale can be interpreted as the result of a balance of the attractive interactions between the amino hydrogen atoms and the oxygen atoms of the sulfonyl group (which can occur only in the eclipsed configuration), and the weak attractive interactions between the sulfonyl oxygen atoms and the methyl group, which occur in conformers *a* and *b*.

In OTS, the methyl internal rotation pathway was explored by varying the corresponding dihedral angle by 10° steps, whereas all the other internal coordinates were freely optimized; the internal rotation barrier (*V*_3_) is reported in the table in Section 2.2.4 below.

### 2.2. Rotational Spectra

The rotational spectra of three compounds, BSA, OTS and PTS, were initially recorded in the 59.6–74.4 GHz frequency range by Stark modulated Free-Jet Absorption Millimeter Wave (FJ-AMMW) spectroscopy. Guided by the theoretical values of the spectroscopic constants, all the spectra were assigned in this frequency range, leading to the determination of rotational constants, centrifugal distortion constants and the barrier hindering the rotation of the methyl group in OTS. Additional experimental measurements were performed in the 2–8 GHz frequency range by Chirped-Pulse Fourier Transform MicroWave (CP-FTMW) spectroscopy. The lower working frequency and the superior resolving capacity of this spectrometer enabled the observation of the hyperfine structures due to the ^14^N quadrupolar nucleus, and thus the determination of the ^14^N nuclear quadrupole coupling constants (*χ*_gg_, *g* = *a*, *b*, *c*). Since the ^14^N nucleus has a spin quantum number greater than 1/2 (*I* = 1), it has a non-spherical charge distribution, and thus a non-vanishing nuclear quadrupole moment (*Q* = 20.44(3) mb). The interaction between *Q* and the electric field gradient of the molecule (*q*_gg_, *g* = *a*, *b*, *c*) at the nucleus provides a mechanism through which the nuclear angular momentum (*I*) and the molecular rotational angular moment (*J*) interact, producing a splitting of the rotational transitions. The analysis of these patterns yields the values of the nuclear quadrupole coupling constants (*χ*_gg_ = *Qq*_gg_e, e = electron charge), which are extremely sensitive to the orientation of the group carrying the quadrupolar nucleus.

#### 2.2.1. The Rotational Spectrum of BSA

**Figure 2:**
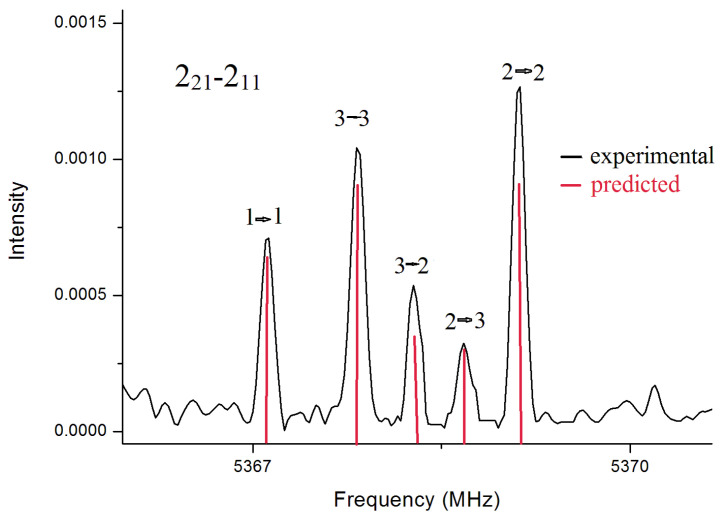
^14^N quadrupole hyperfine structure for the transition 2_2,1_-2_1,1_ of BSA.

In the spectrum for BSA, rotational transitions belonging to only one conformer were identified. Overall, in the two frequency ranges, we assigned *a*-type and *c*-type transitions with lower *J* up to 31, and *K*_a_ up to 18. A global fitting of all transition lines was carried out with Pickett’s SPFIT program [21,22], using a semirigid rotor Hamiltonian in the *S*-reduction and *I*^r^ representation, supplemented with an additional term to account for the nuclear quadrupole coupling contribution. The Hamiltonian was set up in the coupled basis set *I + J = F* and diagonalized in blocks of *F*. The energy levels involved in each transition are thus labelled with the quantum numbers *J*, *K_a_*, *K_c_* and *F*. A depiction of the ^14^N quadrupole hyperfine structure observed for the transition 2_2,1_←2_1,1_ is given in Figure 2 To obtain supplemental structural information, the rotational spectra of its deuterated isotopologues were also recorded in the frequency range 59.6–74.4 GHz. The mono- and bi-deuterated species of the amino group, ND and ND_2_ respectively, were assigned. Measured transition lines were fitted to the Watson’s *S*-reduced semirigid asymmetric rotor Hamiltonian, achieving the spectroscopic constants. The measured frequencies for BSA are reported as Supplementary Material (Table S1) and the spectroscopic parameters are reported in Table 2.

#### 2.2.2. Conformational Assignment and Structure of BSA

The experimental rotational constants were in good agreement with those calculated for the predicted conformers of BSA (Table 2). In addition, the lack of *μ*_b_-transitions confirmed the presence of a symmetry plane along the *a* and *c* axes. Due to the structural similarities between the calculated conformers, which differ only in the position of the amine hydrogen atoms, an unambiguous assignment of the rotational spectrum through a comparison of the experimental and theoretical rotational constants was not possible. Structural identification was provided by the ^14^N nuclear coupling constants, because these parameters critically depend on the electronic environment around the ^14^N nucleus and the principal inertial axis system. A comparison between the experimental and calculated quadrupole coupling constants (Table 2) led to the assignment of the experimental rotational spectrum to the eclipsed conformer (BSA1).

In addition, supplemental information on the structural assignment was obtained from the determined rotational constants for the isotopologues ND and ND_2_ of BSA. Using Kraitchman’s equations and uncertainties estimated according to Costain’s rule settings implemented in the KRA program [23,24], the substitution coordinates for the amino group hydrogen atoms were calculated. The obtained *r*_s_ coordinates were compared to the theoretical coordinates *r*_e_ of BSA1 and BSA2 (Table 3), and these confirmed the assignment of the observed species to BSA1.

#### 2.2.3. The PTS Rotational Spectrum

In the PTS spectrum, *a*-type and *c*-type transitions with *J* up to 31 and *K*_a_ up to 14 were identified. The fitting was accomplished using Watson’s S-reduced semirigid rotor Hamiltonian, as described before for BSA. The agreement between experimental and calculated rotational constants, and the lack of observation of *b*-type transitions, indicated the presence of a *C*_s_ symmetry, suggesting that the observed conformer was one of the calculated structures reported in Table 4. For PTS, as for BSA, the determination of the ^14^N quadrupole coupling constants allowed discrimination between PTS1 and PTS2. Although an ambiguity seemed to remain regarding the assignment to PTS1a or PTS1b, examining the behavior of the methyl group in similar compounds, such as the C_2v_ molecules toluene and *p*-nitrotoluene, allowed us to observe that the methyl group undergoes almost free rotation in the molecule, the barriers being *V*_6_ = 4.83783617(94) cm^−1^ [25] and *V*_6_ = 12.5(22) cm^−1^ [26], respectively, with the vibrational ground state lying above the barrier, making it impossible to localize the hydrogen atoms in a unique position. The same description can be applied to PTS, despite it having two predicted minima. Indeed, the rotation of the methyl group is hindered by a small barrier, which was calculated by Petrov et al. [17] to be 0.126 kJ/mol (10.5 cm^−1^). In this case, generally only the lowest lying torsional state is observed in the cold environment of a free jet expansion, especially if the overall intensity of the spectrum is low, such as in the case of the sulfa-drugs. Accordingly, no additional lines due to the internal rotation of the methyl group were observed in the spectrum of PTS. The measured frequencies for PTS are reported as Supplementary Material (Table S2) and the spectroscopic parameters are reported in Table 4.

#### 2.2.4. The OTS Rotational Spectrum

The spectrum of OTS in the frequency range 59.6 to 74.4 GHz showed splitting of the rotational transitions in two components, A and E, due to the methyl internal rotation coupled with the overall molecular rotation. A first assignment was performed for the component A, which followed the usual selection rules for a pseudo-rigid rotor, obtaining a preliminary set of experimental rotational constants. This fitting was carried out using Watson’s *S*-reduced semirigid rotor Hamiltonian, implemented in the SPFIT program [21,22].

The analysis of the hyperfine structure due to the methyl rotation was performed by the XIAM program developed by Hartwig and Dreizler [27]: a software program that allows the simultaneous analysis of internal rotation and nuclear quadrupole couplings. The input data were the rotational constants determined previously, the parameters of internal rotation such as the potential barrier (*V*_3_), and the structural parameters calculated for the most stable species, namely OTS1. From the analysis, the rotational and quartic centrifugal distortion constants and *V*_3_ were obtained, while the moment of inertia of the top and its orientation were fixed as the B3LYP calculated values. Additional measurements in the range 2–8 GHz allowed the determination of the ^14^N quadrupole coupling constants. Overall *a-*type and *c*-type transitions, with *J* up to 31 and *K*_a_ up to 18, were assigned. The measured frequencies for OTS are reported as Supplementary Material (Table S3), and the spectroscopic parameters are listed in Table 5.

The *V*_3_ height was determined to be 6.356(6) kJ/mol (531.4(5) cm^−1^). Comparisons with other *ortho*-methyl substituted benzenes show that this value is between those of *o*-fluorotoluene (2.7 kJ/mol [28]), *anti*-*o*-cresol (4.426(1) kJ/mol) and *syn*-*o*-cresol (7.91(5) kJ/mol [29]), while it is close to the value of *o*-chlorotoluene (5.580(5) kJ/mol [30]).

The rotational constants determined for OTS are in agreement with those of the conformational species reported in Table 5. However, the similarity between the rotational constants of the four predicted conformers does not allow an immediate structural identification. The observation of *μ*_c_-transitions in the spectrum made it possible to exclude OTS1b and OTS2b. If the ^14^N quadrupole coupling constants are compared with those calculated for OTS1a and OTS2a, the assigned species must be OTS1a. OTS1a was also consistently predicted to be the lowest-energy conformation by both the B3LYP and MP2 theoretical methods. The superior stability of OTS1 can be ascribed to a weak attractive interaction between the nitrogen lone pair and the methyl hydrogen atoms.

#### 2.2.5. The SUA Rotational Spectrum

The rotational spectrum of SUA was observed using the CP-FTMW spectrometer due to its higher sensitivity. Its low overall intensity probably prevented its observation using the FJ-AMMW technique. Several rotational transitions showing nuclear quadruple coupling hyperfine structure with lower *J* ranging from 0 to 6 and *K*_a_ ranging from 0 to 2 were observed and fitted with Pickett’s SPFIT program [21,22], using a semirigid rotor Hamiltonian in the *S*-reduction and *I*^r^ representation, supplemented with an additional term to account for the nuclear quadrupole coupling contribution. The measured frequencies for SUA are reported as Supplementary Material (Table S4) and the spectroscopic parameters are reported in Table 6.

Based on the comparison of the rotational constants alone, it was not possible to assign the observed spectrum to a single calculated structure. We again relied on the experimental nuclear quadrupole coupling constants. In this molecule, there are two sets of nuclear quadrupole coupling constants: one referring to the nitrogen atom of the amino group of the sulfonamide group (N1 in Table 6), and the other referring to the nitrogen atom of the amino group in the *para*-position (N2). For the fitting procedure, the symmetric coupling scheme between the angular momenta *I* = *I*_1_+ *I*_2_ and *F = I + J* was used, thus the quantum states were labelled *J*, *K_a_*, *K_c_*, *I* and *F*. Comparing the values for N1, we can rule out SUA2a and SUA2b. Thus, SUA also prefers to adopt a conformation in which the sulfonamide group is in an eclipsed configuration with respect to the amino group. Regarding the orientation of the *para*-amino groupand, considering that the SUA1a conformer is predicted to be the global minimum by both computational methods, we can assign the observed species as SUA1a. The fact that the second conformer SUA1b was not observed was probably related to the low inversion barrier of the amino group, which allows the higher energy conformer to relax to the global minimum.

## 3. Materials and Methods

Commercial samples of BSA, OTS, PTS and SUA were purchased from Alfa Aesar and used as received. BSA (C_6_H_7_NO_2_S, 157.19 g/mol, 98%), OTS (C_7_H_9_NO_2_S, 171.22 g/mol, 99%), PTS (C_7_H_9_NO_2_S, 171.22 g/mol, 98%) and SUA (C_6_H_8_N_2_O_2_S, 172.21 g/mol, 98%) are white crystalline solids at ambient conditions. Their melting points are 151–154 °C, 156 °C, 136–139 °C and 163 °C, respectively. The spectra of BSA, OTS and PTS were recorded by two different spectrometers over two different frequency ranges.

At the University of Bologna, the BSA, OTS and PTS spectra were recorded in the 59.6 to 74.4 GHz frequency range by a Free-Jet Stark modulated Absorption Millimeter Wave spectrometer (FJ-AMMW) [31,32,33]. The resolution and the estimated accuracy of the frequency measurements were approximately 300 kHz and 50 kHz, respectively. To obtain a suitable concentration of the samples, the substances were heated: BSA to 140–150 °C; OTS and PTS to 130–140 °C. Successively, the samples were expanded in argon from a pressure of 20 kPa to about 0.5 Pa, through a 0.3 mm diameter pinhole nozzle. The deuterated isotopologues species for BSA were obtained by passing D_2_O in argon over the heated sample.

At King’s College London, the BSA, OTS, PTS and SUA spectra were recorded in the 2–8 GHz frequency range using a pulsed jet chirped-pulse Fourier transform microwave spectrometer (CP-FTMW) [34,35,36]. All compounds were vaporized by heating, with optimal spectral signals obtained with temperatures of 169 °C (BSA), 165 °C (OTS and PTS) and 167 °C (SUA). The compounds were seeded in neon gas at backing pressures of approximately 5 bar. Typical molecular pulses of 1000 μs were used to produce the supersonic jet in the vacuum chamber. Four microwave chirped-pulses of 4 μs length, spaced 30 μs apart, were applied per molecular pulse. The first chirped-pulse had a delay of 1100 μs with respect to the start of the molecular pulse. Molecular relaxation signals were collected for 20 μs after each microwave pulse using a digital oscilloscope, and converted into the frequency domain through a fast Fourier-transform algorithm. The estimated accuracy of frequency measurements was 10 kHz. Final spectra had 2.4 M FIDs (BTS), 1.2 M FIDs (PTS), 1.0 M FIDs (OTS) and 874 k FIDs (SUA).

PTS was also vaporized by laser ablation, which yielded a more intense spectrum than heating. For the laser ablation we used a ps Nd:YAG laser in the third harmonic with a 9–10 mJ/pulse, and neon backing pressure of 13 bar. The delay between the molecular pulse and the laser pulse was 625 μs. The final spectra obtained from laser ablation had 235 k FIDs.

## 4. Conclusions

In this work, the conformational preferences of the compounds BSA, PTS, OTS and SUA were analyzed using their rotational spectra recorded by means of FJ-AMMW and CP-FTMW spectrometers working in the 59.6–74.4 GHz and 2–8 GHz frequency ranges, respectively. Their rotational and centrifugal distortion, and their ^14^N quadrupole coupling constants, were determined. The latter were instrumental in achieving a conclusive identification of the preferred conformers for all species. For OTS, the hindering barrier of the methyl group rotation was also determined. The experimentally derived *V*_3_ energy barrier for the rotation of methyl group in OTS (6.157(6) kJ/mol) is in good agreement with the calculated B3LYP/6-311++G** barrier (5.646 kJ/mol), but 45 times higher than the corresponding B3LYP calculated barrier for PTS. The large differences in energy values are a result of the interaction between the methyl group and the sulfonamide group. In OTS, rotation is hindered, while there is almost free methyl rotation in PTS. The calculated value, *V*_3_ = 0.126 kJ/mol (Petrov et al. [17]), is very similar to that found for toluene (*V*_6_ = 0.057873430(6) kJ/mol [37]), which is a prototype for free methyl internal rotation.

Our study showed that weak intramolecular interactions are able to change the conformational preferences of the pharmacophore of benzensulfonamides. In the compounds BSA, PTS and SUA, where there are no groups close to the sulfonamide tail, the conformational behavior is similar. In these cases, the amino group lies perpendicular to the aromatic plane, and a plane of symmetry is present. The symmetry was also reflected in the very similar values of the *χ*_bb_ nuclear quadruple coupling constants, which were equal, within experimental uncertainty, in PTS and BSA. In contrast, in OTS the presence of a methyl group in *ortho* to the sulfonamide tail results in the establishment of a weak attractive interaction between the nitrogen lone pair and the methyl hydrogen atoms. This, in turn, changes the arrangement of the amino group, which for OTS is in a *gauche* configuration with respect to the benzene ring. Interestingly, the H atoms of the amino group retain their eclipsed configuration with the O atoms of the sulfonyl group. The eclipsed configuration of the hydrogen atoms of the amino groups and the oxygen atoms of the sulfonyl group was maintained for all the studied species. The nature of the stabilization of the eclipsed conformations, with respect to the staggered ones, could result from an electronic effect in which the eclipsed conformations tend to minimize the total dipole moment. Some authors attribute the stabilization to the existence of electrostatic attractive interactions between O and amino H atoms (see, for example, [38]), but this seems unlikely, given the orientation of the O lone pairs of the sulfone group with respect to the amino H atoms.

One of the questions in the study of drug molecules is the extent to which the inherent conformations are modified by interactions with the protein target, or by crystal forces, in those cases where the X-ray crystal structures have been obtained. Analysis of the structures of sulfonamides listed in the Cambridge Structural Dataset found that those with a substituent in *ortho* deviate from the perpendicular orientation of the sulfonyl group with respect to the benzene ring, and they show, on average, a configuration where the S=O bond lies on the benzene plane [38]. In light of our results, the main conformational features observed for the isolated sulfonamides appear to be retained when subjected to crystal forces.

A search of the PDB database showed that BSA, SUA and OTS are present in several crystals whose structure has been studied by X-ray techniques. The measured ∠CCSN torsion angles are listed and visualized in the Supplementary Material (Figure S1). Here, the first four letters correspond to the PDB-ID of the macromolecular structure, and the following three letters identify the PDB-ID of the ligand. In most of these structures, the ∠CCSN angle assumes all values from 0 to 90° with respect to the benzene ring. These twisted configurations have an energy cost with respect to the minimum energy of the bare sulfonamides, and this must be overcome by the stabilising interactions of the residues at the binding site of the protein target. Now that the lowest energy structures in the gas phase have been established, the energy costs associated with a twisted structure can be more accurately estimated, without any concerns regarding crystal lattice effects influencing the configuration of the isolated molecule.

In summary, our results show that rotational spectroscopy is an ideal tool to identify molecular conformations without ambiguity, and to reveal the intramolecular interactions involved. The results for the sulfonamide molecules presented here can be utilized as the basis for further studies on this class of drugs, for example examining their complexes with water and with mimics of amino acid residues at relevant receptor sides, to obtain information on their intermolecular interactions.nteractions at relevant receptor sites.

## Figures and Tables

**Table 1:** Theoretical conformers: sketch, labeling, relative energies and electric dipole moment (Δ*E*_e_/Δ*E*_0_ [kJ/mol]; *µ* [D]). First row reports the B3LYP/6-311++G** values, second row reports the MP2/6-311++G** values.

Eclipsed		Staggered	
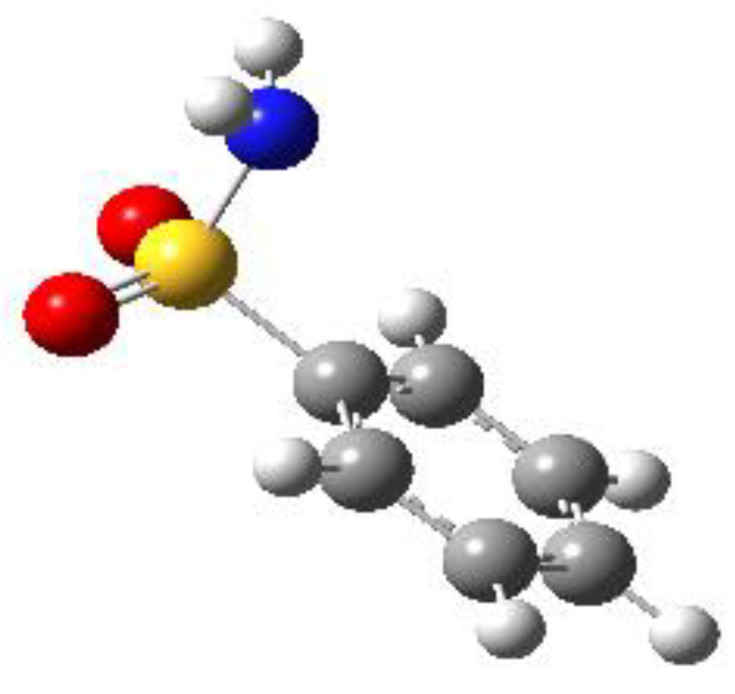		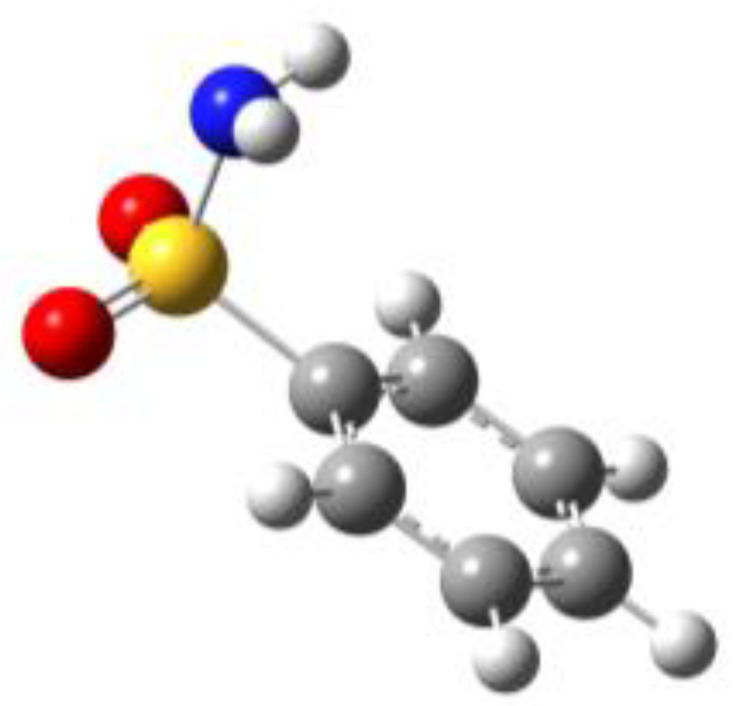	
**BSA-1** 0^a^/0^b^; 4.0 0^c^/0^d^; 3.8		**BSA-2** 2.75/3.56; 6.1 1.24/5.00; 5.9	
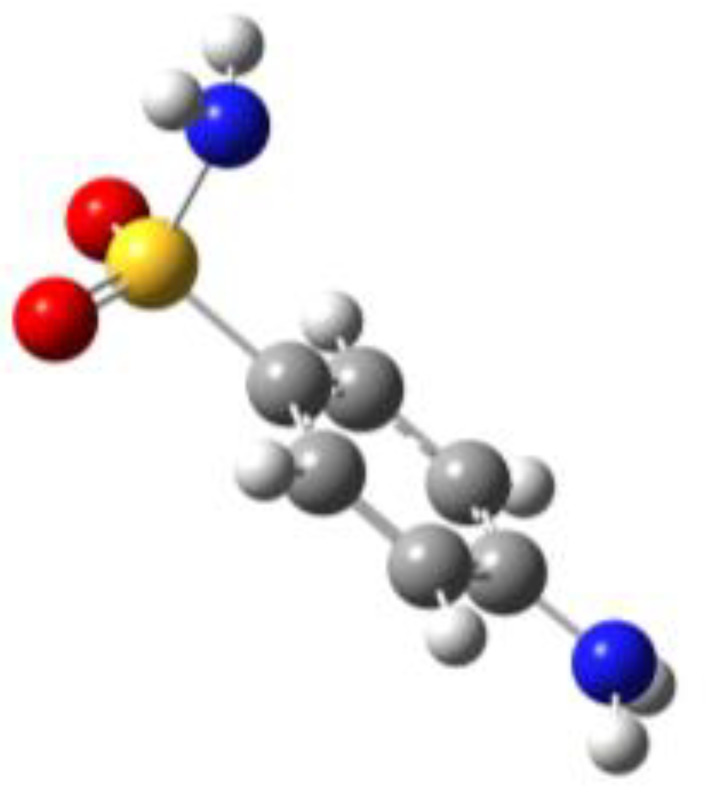	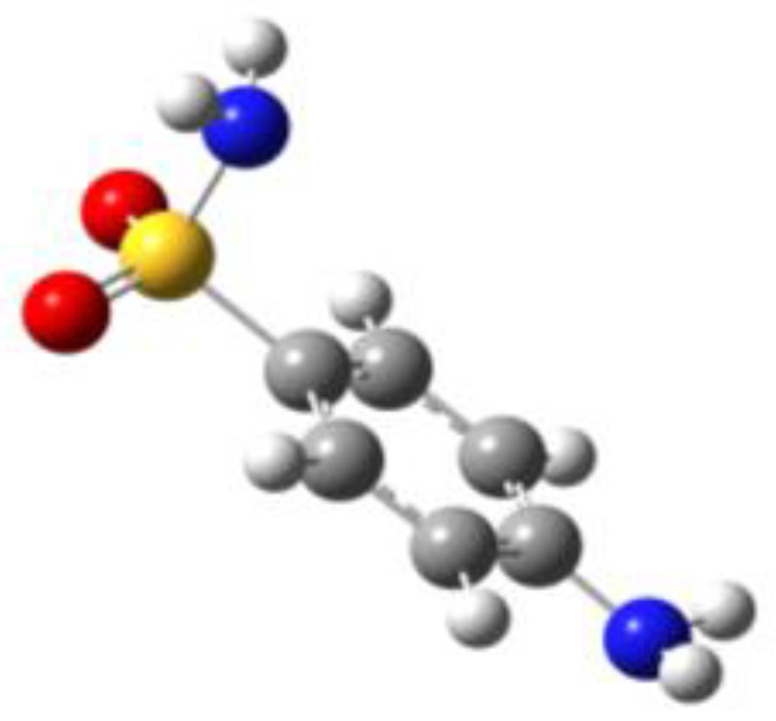	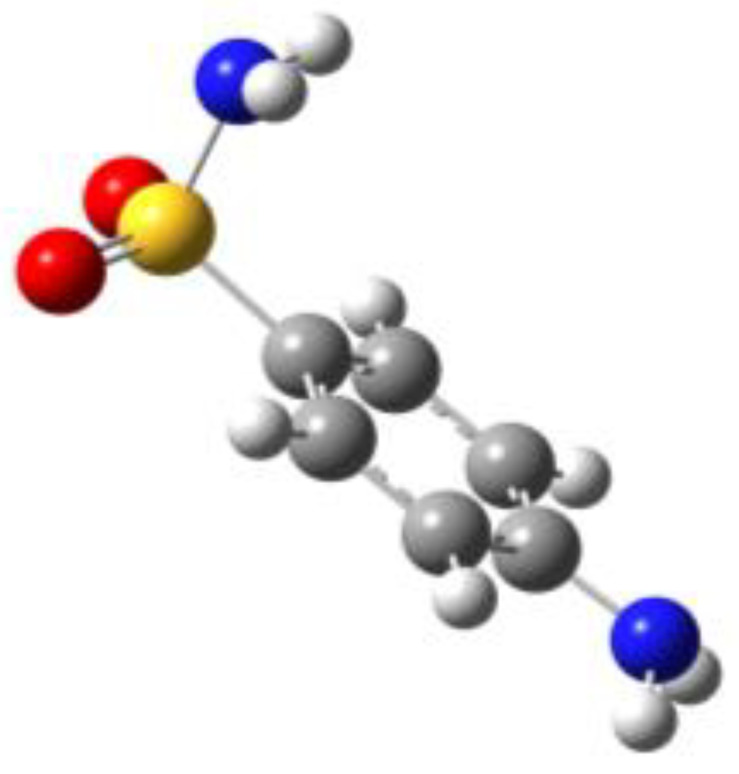	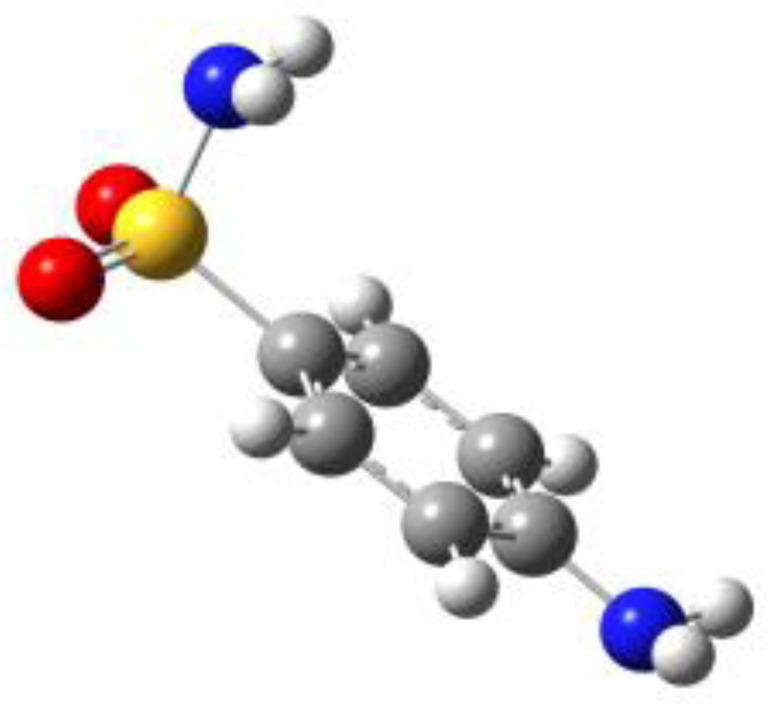
**SUA-1a** 0^e^/0^f^; 5.2 0^g^/0^h^; 4.5	**SUA-1b** 0.29/0.12; 6.0 0.35/−0.28; 5.6	**SUA-2a** 2.26/3.30; 7.6 0.31/1.64; 6.9	**SUA-2b** 2.98/3.77; 8.3 1.12/2.59; 7.8
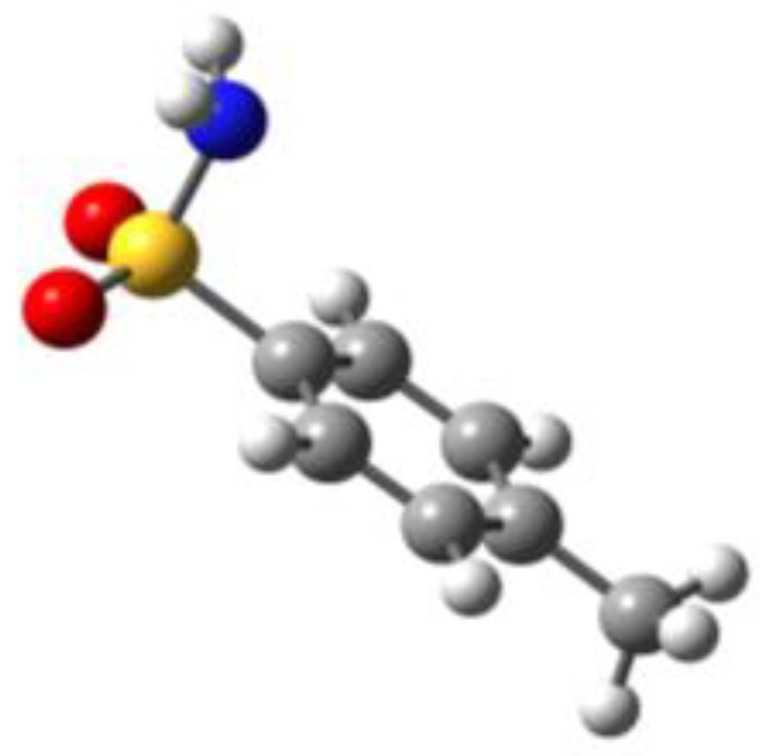	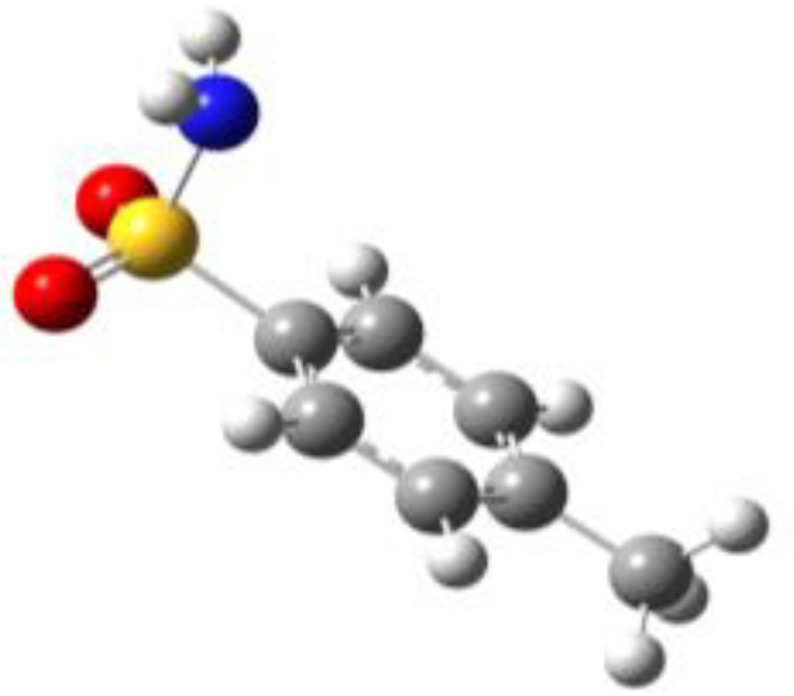	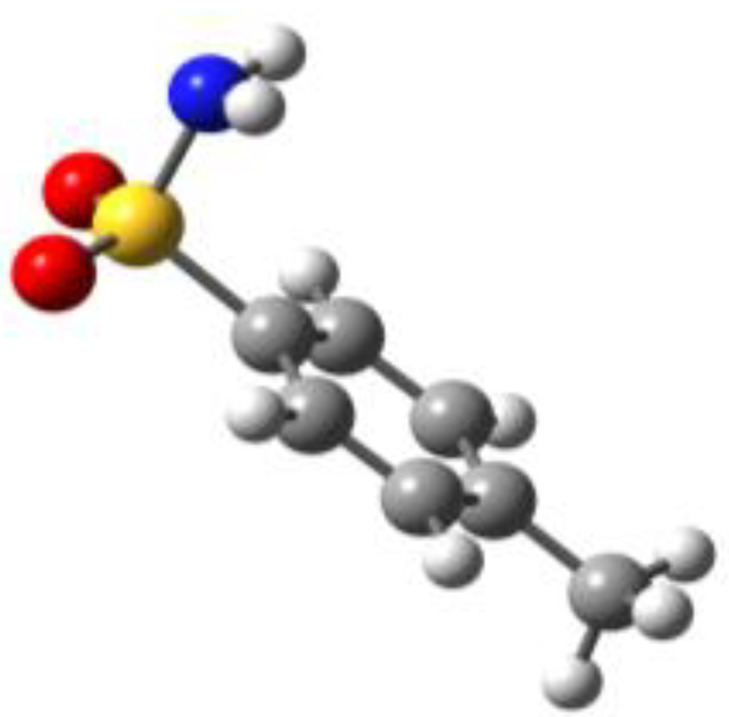	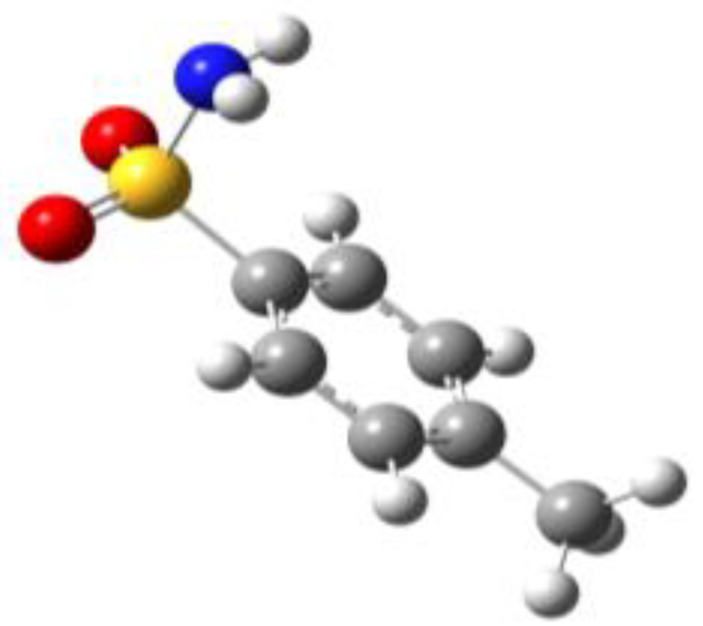
**PTS-1a** 0^i^/0^j^; 4.4 0^k^/0^l^; 4.2	**PTS-1b** 0.55/0.32; 4.5 0.03/0.09; 4.2	**PTS-2a** 2.69/3.50; 6.6 0.79/2.57; 6.4	**PTS-2b** 2.71/3.53; 6.7 0.83/2.65; 6.4
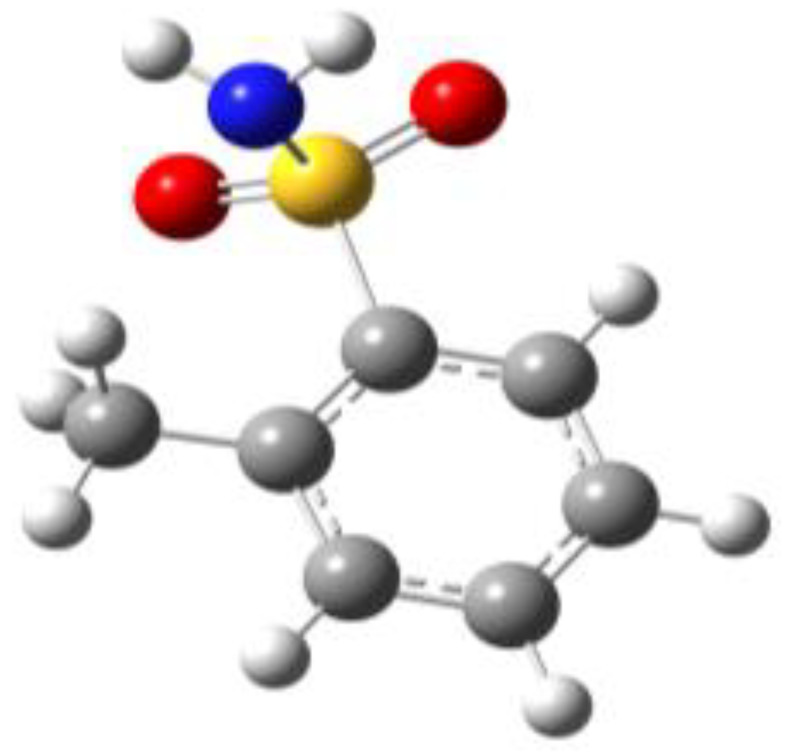	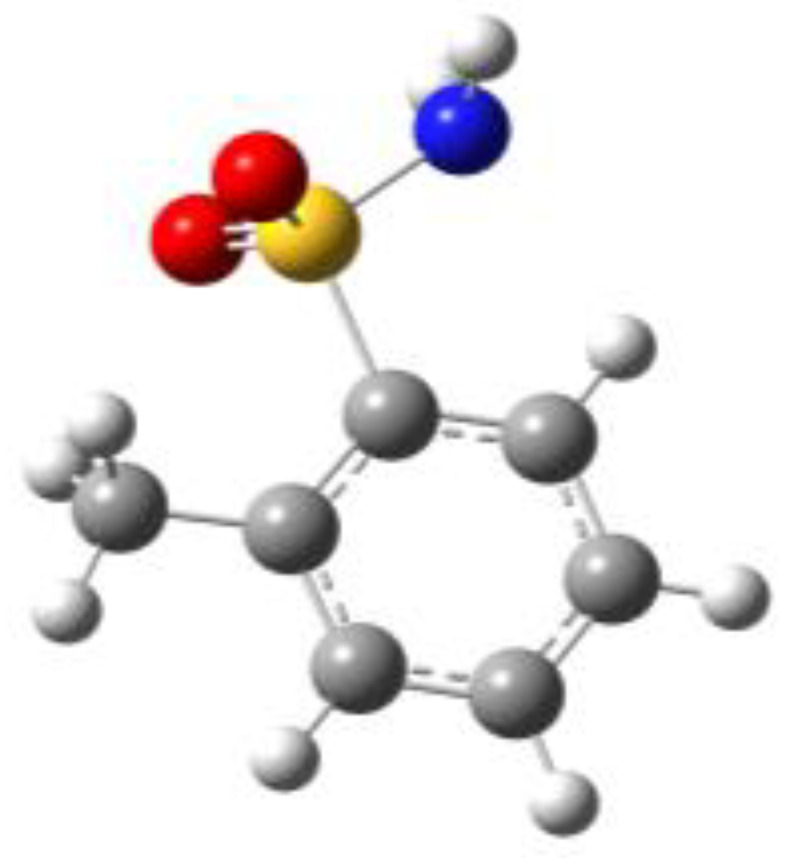	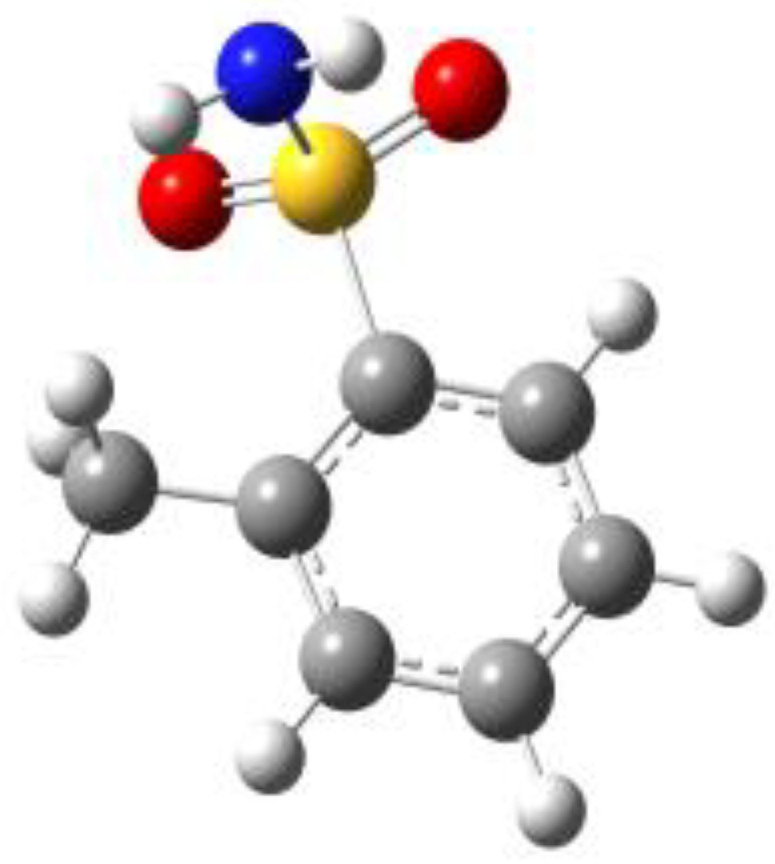	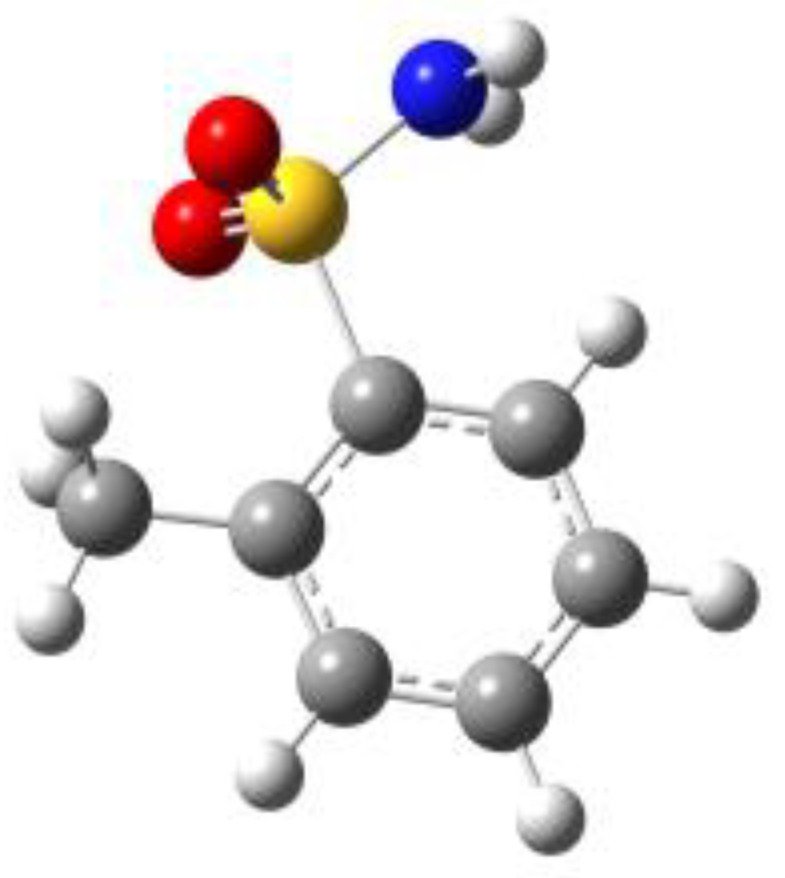
**OTS-1a** 0^m^/0^n^; 4.0 0^o^/0^p^; 3.8	**OTS-1b** 5.14/4.39; 3.4 5.42/5.80; 3.2	**OTS-2a** 6.80/6.96; 5.9 5.46/6.70; 5.7	**OTS-2b** 13.6/12.5; 5.1 12.5/12.8; 5.1

^a^*E*_e_ = −836.327624 au, ^b^*E*_0_ = −836.200943 au, ^c^*E*_e_ = −834.583358 au, ^d^*E*_0_ = −834.457246 au, ^e^*E*_e_ = −891.707242 au, ^f^*E*_0_ = −891.564105 au, ^g^*E*_e_ = −889.816853 au, ^h^*E*_0_ = −889.672869 au, ^i^*E*_e_ = −875.656160 au, ^j^*E*_0_ = −875.502398 au, ^k^*E*_e_ = −873.784244 au, ^l^*E*_0_ = −873.629671 au, ^m^*E*_e_ = −875.654099 au, ^n^*E*_0_ = −875.499644 au, ^o^*E*_e_ = −873.784399 au, ^p^*E*_0_ = −873.627236 au.

**Table 2:** Theoretical and experimental spectroscopic parameters of BSA.

	Theoretical Results		Experimental Results		
	BSA1	BSA2	BSA-NH_2_	BSA-NDH	BSA-ND_2_
*A* (MHz)	2571/2572 ^a^	2574/2575	2627.657(1) ^b^	2577.4061(7)	2531.2916(7)
*B* (MHz)	822/832	820/830	838.2808(3)	826.662(6)	815.30(1)
*C* (MHz)	718/725	716/722	730.4887(3)	724.128(5)	717.64(1)
*D*_J_ (kHz)	0.035/0.048	0.029/0.027	0.0426(2)	[0.043] ^c^	[0.043]
*D*_JK_ (kHz)	0.122/0.254	0.106/0.101	0.098(2)	[0.098]	[0.098]
*D*_K_ (kHz)	−0.013/−0.160	0.003/0.0002	-	-	-
*d*_1_ (kHz)	−0.003/−0.003	−0.003/−0.003	−0.0024(2)	[−0.0024]	[−0.0024]
*d*_2_ (kHz)	0.008/0.011	0.005/0.004	0.01201(6)	[0.01201]	[0.01201]
*χ*_aa_ (MHz)	−2.92/−2.87	−4.97/−4.65	−2.62(1)	-	-
*χ*_bb_ (MHz)	1.61/1.56	1.78/1.71	1.37(7)	-	-
*χ*_cc_ (MHz)	1.32/1.31	3.19/2.95	1.25(7)	-	-
N ^d^	-	-	154	45	27
σ ^e^ (kHz)	-	-	61	69	59
*μ*_a_ (D)	2.61/2.42	4.83/4.76	Y ^f^	N	N
*μ*_b_ (D)	0/0	0/0	N	N	N
*μ*_c_ (D)	3.01/2.94	3.72/3.62	Y	Y	Y
Δ*E* (kJ/mol)	0	2.75/1.24	-	-	-
Δ*E*_0_ (kJ/mol)	0	3.56/5.00	-	-	-

^a^ The two values refer to B3LYP and MP2 results. In both cases the 6-311++G** basis set was used. ^b^ Standard error in parentheses in the units of the last digit. ^c^ Values in squared brackets are fixed to those of the parent species. ^d^ Number of transitions. ^e^ Standard deviation of the fit. ^f^ Y or N indicates whether this transition type has been observed or not.

**Table 3:** Theoretical (B3LYP/6-311++G**) and experimental principal axes system coordinates of the amino hydrogen atoms in BSA.

	exp. NH ^a^	exp. NH ^b^		BSA1	BSA2
|*a*_s_| (Å)	2.313(1) ^c^	2.362(2)	*a*_e_ (Å)	−2.4144	−1.7345
|*b*_s_| (Å)	0.813(3)	0.816(5)	*b*_e_ (Å)	−0.8428	−0.8479
|*c*_s_| (Å)	1.768(1)	1.715(2)	*c*_e_ (Å)	−1.7369	−1.9853

^a^ Parent species NH_2_ ^b^ Parent species ND_2_^c^ Costain’s errors expressed in units of the last decimal digit.

**Table 4:** Theoretical and experimental spectroscopic parameters of PTS.

	Theoretical Results				Experimental Results
	PTS1a	PTS1b	PTS2a	PTS2b	PTS
*A* (MHz)	2538/2538 ^a^	2539/2537	2539/2540	2540/2538	2634.331(3) ^b^
*B* (MHz)	554/560	554/560	553/558	553/558	563.2070(5)
*C* (MHz)	505/509	505/509	504/508	504/508	512.8048(5)
*D*_J_ (kHz)	0.014/0.012	0.014/0.013	0.014/0.010	0.010/0.009	0.019(1)
*D*_JK_ (kHz)	0.094/0.138	0.092/0.152	0.087/0.070	0.077/0.069	0.044(8)
*D*_K_ (kHz)	0.044/0.015	0.039/−0.023	0.050/0.051	0.051/0.060	0.101(8)
*d*_1_ (kHz)	−0.001/−0.001	−0.001/−0.001	−0.001/−0.001	−0.001/−0.001	−0.025(4)
*d*_2_ (kHz)	0.003/0.002	0.003/0.003	0.003/0.001	0.001/0.000	-
*χ*_aa_ (MHz)	−2.95/−2.90	−2.94/−2.93	−4.99/−4.66	−4.98/−4.67	−2.63(1)
*χ*_bb_ (MHz)	1.62/1.56	1.62/1.57	1.78/1.70	1.78/1.78	1.35(2)
*χ*_cc_ (MHz)	1.33/1.34	1.32/1.37	3.21/2.96	3.20/2.97	1.27(2)
N ^c^	-	-	-	-	89
σ ^d^ (kHz)	-	-	-	-	41
*μ*_a_ (D)	−3.00/−3.01	−3.27/−3.01	−5.55/−5.40	−5.54/−5.41	Y ^e^
*μ*_b_ (D)	0/0	0/0	0/0	0/0	N
*μ*_c_ (D)	−2.87/−2.87	−2.95/−2.94	−3.70/3.45	−3.63/−3.50	Y
Δ*E* (kJ/mol)	0	0.55/0.03	2.69/0.79	2.72/0.83	-
Δ*E*_0_ (kJ/mol)	0	0.32/0.09	3.50/2.57	3.53/2.65	-

^a^ The two values refer to B3LYP and MP2 results. In both cases the 6-311++G** basis set was used. ^b^ Standard error in parentheses in the units of the last digit ^c^ Number of transitions. ^d^ Standard deviation of the fit. ^e^ Y or N indicates whether this transition type has been observed or not.

**Table 5:** Theoretical and experimental spectroscopic parameters of OTS.

	Theoretical Results				Experimental Results ^a^
	OTS1a	OTS1b	OTS2a	OTS2b	OTS
*A* (MHz)	1727/1730 ^b^	1729/1733	1722/1723	1735/1738	1755.075(2) ^c^
*B* (MHz)	812/822	809/818	807/818	805/813	827.0307(5)
*C* (MHz)	628/634	625/631	624/630	624/629	637.1196(5)
*D*_J_ (kHz)	0.021/0.021	0.051/0.023	0.024/0.021	0.036/0.023	0.0207(8)
*D*_JK_ (kHz)	0.051/0.053	−0.001/0.046	0.044/0.047	0.024/0.042	0.058(4)
*D*_K_ (kHz)	0.003/0.000	0.026/0.006	0.009/0.008	0.016/0.009	0.009(3)
*d*_1_ (kHz)	−0.003/−0.004	−0.003−0.003	−0.004/−004	−0.004/−0.003	0.013(2)
*d*_2_ (kHz)	0.002/0.002	0.017/0.004	0.004/0.002	0.010/0.000	-
*χ*_aa_ (MHz)	−2.05/−1.92	−2.36/−2.12	−4.88/−4.58	−4.68/−4.35	−2.030(9)
*χ*_bb_ (MHz)	−0.32/−0.26	0.73/0.51	1.87/1.75	2.72/2.49	0.442(13)
*χ*_cc_ (MHz)	2.37/2.18	1.63/1.62	3.01/2.84	1.96/1.86	1.587(13)
*V*_3_ (kJ/mol)	5.646 ^d^	-	-	-	6.157(6)
N ^e^	-	-	-	-	135
σ ^f^ (kHz)	-	-	-	-	44
*μ*_a_ (D)	2.47/2.30	−2.08/−1.88	4.54/4.51	−3.82/−3.80	Y ^g^
*μ*_b_ (D)	1.25/0.95	2.64/2.54	1.64/1.22	3.44/3.29	Y
*μ*_c_ (D)	−2.91/−2.86	0.02/0.70	−3.40/−3.28	0.02/0.66	Y
Δ*E* (kJ/mol)	0	5.14/5.42	6.80/5.46	13.6/12.5	-
Δ*E*_0_ (kJ/mol)	0	4.39/5.80	6.96/6.70	12.5/12.8	-

^a^ The following parameters were fixed as the average value between B3LYP/6-311++G** and MP2/6-311++G** data of the OTS1a species in the fit: F_0_ = *h*^2^/(2I_α_) = 160 GHz, δ = −1.125 rad, ε = 0.011 rad, where I_α_ is moment of inertia of the top about its symmetry axis (i), δ is the angle between the projection of the internal rotation axis *i* onto the ab plane axis and the a principal axis, ε is the angle between the projection of the internal rotation axis *i* onto the *bc* plane and the *b* principal axis. ^b^ The two values refer to B3LYP and MP2 results. In both cases the 6-311++G** basis set was used. ^c^ Standard error in parentheses in the units of the last digit. ^d^ B3LYP/6-311++G** value. ^e^ Number of transitions. ^f^ Standard deviation of the fit. ^g^ Y or N indicates whether this transition type has been observed or not.

**Table 6:** Theoretical and experimental spectroscopic parameters of SUA.

	Theoretical Results				Experimental Results
	SUA1a	SUA1b	SUA2a	SUA2b	SUA
*A* (MHz)	2554/2556 ^a^	2554/2556	2555/2557	2555/2557	2609.4(1) ^b^
*B* (MHz)	556/561	556/561	555/560	555/560	565.0243(4)
*C* (MHz)	506/510	506/510	505/508	505/508	513.4812(4)
*D*_J_ (kHz)	0.017/0.010	0.032/0.033	0.010/0.010	0.010/0.001	0.037(4)
*D*_JK_ (kHz)	0.137/0.105	0.230/0.631	0.076/0.073	0.076/0.072	-
*D*_K_ (kHz)	0.001/0.025	−0.106/0.519	0.063/0.058	0.063/0.058	-
*d*_1_ (kHz)	−0.001/−0.001	−0.001/−0.001	−0.001/−0.001	−0.001/−0.001	-
*d*_2_ (kHz)	0.005/0.002	0.012/0.012	0.001/0.000	0.001/0.000	-
*χ*_aa_ (N1) ^c^ (MHz)	−2.71/−2.37	−2.84/−2.70	−4.97/−4.67	−4.97/−4.67	−2.63(5)
*χ*_bb_ (N1) (MHz)	1.33/0.73	1.62/1.28	1.78/1.70	1.79/1.71	-
*χ*_cc_ (N1) (MHz)	1.37/1.64	1.22/1.42	3.19/2.96	3.18/2.96	-
*χ*_aa_ (N2) (MHz)	2.67/2.53	2.53/2.34	2.69/2.56	2.55/2.35	2.89(5)
*χ*_bb_ (N2) (MHz)	2.22/1.94	2.24/1.95	2.21/1.94	2.24/1.95	-
*χ*_cc_ (N2) (MHz)	−4.89/−4.48	−4.77/−4.29	−4.91/−4.50	−4.79/−4.30	-
N ^d^	-	-	-	-	29
*σ* ^e^ (kHz)	-	-	-	-	16
*μ*_a_ (D)	−4.73/−4.09	−4.68/−3.96	−7.00/−6.41	−6.99/−6.36	Y ^f^
*μ*_b_ (D)	−0.05/0.09	0/0	0/0	0/0	N
*μ*_c_ (D)	−2.15/−1.89	−3.76/−3.94	−2.85/−2.45	−4.45/−4.51	N
Δ*E* (kJ/mol)	0	0.29/0.35	2.26/0.31	2.98/1.12	-
Δ*E*_0_(kJ/mol)	0	0.12/−0.28	3.30/1.64	3.77/2.59	-

^a^ The two values refer to B3LYP and MP2 results. In both cases the 6-311++G** basis set was used. ^b^ Standard error in parentheses in the units of the last digit. ^c^ N1 refers to the amino group of the sulfonamide group while N2 refers to nitrogen in the amino group in the *para*-position with respect to the sulfonamide group. ^d^ Number of transitions. ^e^ Standard deviation of the fit. ^f^ Y or N indicates whether this transition type has been observed or not.

## Data Availability

Not applicable.

## Supplementary Materials

The following are available online at https://www.mdpi.com/article/10.3390/molecules27092820/s1, Table S1. Measured transition frequencies for BSA (MHz). Table S2. Measured transition frequencies for PTS (MHz). Table S3. Measured transition frequencies for OTS (MHz). Table S4. Measured transition frequencies for SUA (MHz). Figure S1. ∠CCSN torsion angle values found in PDB for BSA, SUA and OTS.

## Sample Availability

Commercial.
