# Supplementary material for: The Shapes of Sulfonamides: A Rotational Spectroscopy Study
Source: Molecules. 2022 Apr 28;27(9):2820. doi: 10.3390/molecules27092820 (PMC9101976; doi:10.3390/molecules27092820)
Supplement: Supplementary file 1 [file molecules-27-02820-s001.zip › molecules-1698999-supplementary.pdf]

Article

The Shapes of Sulfonamides: A Rotational Spectroscopy Study

Annalisa Vigorito [1], Camilla Calabrese [1,2], Assimo Maris [1], Donatella Loru [3,4], Isabel Peña [2,3], M. Eugenia Sanz [3] and Sonia Melandri [1,*]

[1] Dipartimento di Chimica "G. Ciamician" dell'Università, via Selmi 2, I-40126 Bologna, Italy; vigo-giff@gmail.com (A.V.); calabrese.cami@gmail.com (C.C.); assimo.maris@unibo.it (A.M.)

[2] Current address: Departamento de Química Física y Química Inorgánica, Facultad de Ciencias—I.U.CINQUIMA,
Universidad de Valladolid, E-47011 Valladolid, Spain; mipencal@uva.es

[3] Department of Chemistry, King's College London, Britannia House, 7 Trinity Street, London SE1 1DB, UK; donatella.loru@desy.de (D.L.); maria.sanz@kcl.ac.uk (M.E.S.)

[4] Current address: Deutsches Elektronen-Synchrotron DESY, Notkestraße 85, 22607 Hamburg, Germany

* Correspondence: sonia.melandri@unibo.it

CONTENTS

Table S1. Measured transition frequencies for BSA (MHz).
Table S2. Measured transition frequencies for PTS (MHz).
Table S3. Measured transition frequencies for OTS (MHz).
Table S4. Measured transition frequencies for SUA (MHz).
Figure S1. ∠CCSN torsion angle values found in PDB for BSA, SUA and OTS

Table S1. Measured transition frequencies for BSA (MHz).

								BSA-NH_2	
J''	K_a''	K_c''	F''	J'	K_a'	K_c'	F'	ν	$\Delta\nu$
2	0	2	2	1	0	1	2	3132.022	-0.006
2	0	2	1	1	0	1	0	3132.175	0.019
2	0	2	3	1	0	1	2	3132.857	-0.012
2	1	2	2	1	1	1	1	3029.083	-0.008
2	1	2	3	1	1	1	2	3029.907	0.004
2	1	2	1	1	1	1	0	3030.741	-0.004
2	1	1	2	1	1	0	1	3244.665	-0.009
2	1	1	3	1	1	0	2	3245.486	-0.005
2	1	1	1	1	1	0	0	3246.292	-0.004
3	0	3	4	2	0	2	3	4687.497	0.001
3	1	3	3	2	1	2	2	4541.559	-0.003
3	1	3	4	2	1	2	3	4541.791	-0.003
3	1	2	3	2	1	1	2	4864.814	0.007
3	1	2	4	2	1	1	3	4865.039	-0.003
3	2	2	3	2	2	1	2	4705.642	-0.004
3	2	2	4	2	2	1	3	4706.487	-0.001
3	2	2	2	2	2	1	1	4706.957	0.001
3	2	1	4	2	2	0	3	4725.323	-0.007
4	0	4	5	3	0	3	4	6228.352	-0.026
4	1	4	4	3	1	3	3	6050.352	0.006
4	1	4	5	3	1	3	4	6050.463	0.014
4	1	3	4	3	1	2	3	6480.743	0.007
4	1	3	5	3	1	2	4	6480.856	0.016
4	2	3	4	3	2	2	3	6271.121	-0.002
4	2	3	5	3	2	2	4	6271.486	0.006
4	2	2	4	3	2	1	3	6317.832	0.001
4	2	2	5	3	2	1	4	6318.195	0.003
5	0	5	6	4	0	4	5	7751.981	0.006
5	1	5	6	4	1	4	5	7555.031	0.014
5	2	4	5	4	2	3	4	7833.185	-0.004
5	2	4	6	4	2	3	5	7833.379	0.004
5	2	3	5	4	2	2	4	7925.034	-0.002
5	2	3	6	4	2	2	5	7925.248	0.020

								BSA-NH_2		BSA-NHD		BSA-ND_2	
J''	K_a''	K_c''	F''	J'	K_a'	K_c'	F'	ν	$\Delta\nu$	ν	$\Delta\nu$	ν	$\Delta\nu$
12	12	1		11	11	1		61220.38	0.031	60055.53	0.02		
12	12	0		11	11	0		61220.38	0.031	60055.53	0.02		
13	12	2		12	11	2				61607.61	0.03	60520.07	0.07
13	12	1		12	11	1				61607.61	0.03	60520.07	0.07
13	13	0		12	12	0		66475.37	-0.037	65209.97	-0.08		
14	11	4		13	10	4		60675.40	-0.021	65209.97	-0.08		
14	11	3		13	10	3		60675.40	-0.021	65209.97	-0.08		
14	12	3		13	11	3		64360.46	-0.115	63159.47	-0.09	62054.10	0.06
14	12	2		13	11	2		64360.46	-0.115	63159.47	-0.09	62054.10	0.06
14	14	1		13	13	1		71730.50	0.079	70364.62	0.07	69110.53	0.02
14	14	0		13	13	0		71730.50	0.079	70364.62	0.07	69110.53	0.02
15	11	5		14	10	5		62245.05	-0.060	61108.45	-0.03	60059.30	0.00
15	11	4		14	10	4		62245.05	-0.060	61108.45	-0.03	60059.30	0.00
15	12	4		14	11	4		65930.57	0.057	64711.50	0.08	63587.91	-0.04

15	12	3	14	11	3	65930.57	0.057	64711.50	0.08	63587.91	-0.04
15	13	3	14	12	3	69615.55	-0.047				
15	13	2	14	12	2	69615.55	-0.047				
15	14	2	14	13	2	73300.54	0.013	66762.04	-0.05	65582.22	-0.09
15	14	1	14	13	1	73300.54	0.013	66762.04	-0.05	65582.22	-0.09
16	10	7	15	9	7	60127.85	0.072				
16	10	6	15	9	6	60127.85	0.072				
16	11	6	15	10	6	63814.48	-0.011	62659.92	0.10	61592.72	-0.02
16	11	5	15	10	5	63814.48	-0.011	62659.92	0.10	61592.72	-0.02
16	12	5	15	11	5	67500.17	-0.089	66263.10	0.01	65121.71	0.02
16	12	4	15	11	4	67500.17	-0.089	66263.10	0.01	65121.71	0.02
16	13	4	15	12	4	71185.46	-0.079				
16	13	3	15	12	3	71185.46	-0.079				
16	14	3	15	13	3					72178.60	0.05
16	14	2	15	13	2					72178.60	0.05
17	10	8	16	9	8	61695.76	-0.108	60605.77	-0.04		
17	10	7	16	9	7	61695.76	-0.108	60605.77	-0.04		
17	11	7	16	10	7					63125.82	-0.01
17	11	6	16	10	6					63125.82	-0.01
17	12	6	16	11	6	69069.75	-0.002	67814.43	-0.10	66655.28	0.06
17	12	5	16	11	5	69069.75	-0.002	67814.43	-0.10	66655.28	0.06
17	13	5	16	12	5	72755.21	-0.109				
17	13	4	16	12	4	72755.21	-0.109				
17	17	0	17	16	2	60799.22	0.022				
17	17	1	17	16	1	60799.22	0.022				
18	5	13	17	2	15	62150.12	-0.043				
18	10	9	17	9	9	63263.22	0.021	62155.19	-0.05	61126.89	-0.08
18	10	8	17	9	8	63263.22	0.021	62155.19	-0.05	61126.89	-0.08
18	11	8	17	10	8	66951.94	-0.029	65761.33	0.04	64658.38	-0.12
18	11	7	17	10	7	66951.94	-0.029	65761.33	0.04	64658.38	-0.12
18	12	7	17	11	7	70638.96	0.039	69365.59	-0.07		
18	12	6	17	11	6	70638.96	0.039	69365.59	-0.07		
18	13	6	17	12	6	74324.82	-0.064				
18	13	5	17	12	5	74324.82	-0.064				
18	17	1	18	16	3	60797.15	0.134				
18	17	2	18	16	2	60797.15	0.134				
18	18	1	18	17	1	64483.95	-0.046				
18	18	0	18	17	2	64483.95	-0.046				
19	5	14	18	2	16	63408.29	0.043				
19	9	11	18	8	11	61134.71	-0.101				
19	9	10	18	8	10	61134.71	-0.100				
19	10	10	18	9	10	64829.60	-0.020	63703.83	0.00	62657.91	0.05
19	10	9	18	9	9	64829.60	-0.020	63703.83	0.00	62657.91	0.05
19	11	9	18	10	9	68519.95	0.085	67311.30	0.06	66190.62	-0.03
19	11	8	18	10	8	68519.95	0.085	67311.30	0.06	66190.62	-0.03
19	12	8	18	11	8	72207.75	0.060	70916.42	0.01		
19	12	7	18	11	7	72207.75	0.060	70916.42	0.01		
19	17	2	19	16	4	60794.60	0.137				
19	17	3	19	16	3	60794.60	0.137				
19	18	1	19	17	3	64481.88	0.049				
19	18	2	19	17	2	64481.88	0.049				
20	4	16	19	1	18	61263.96	-0.016				
20	5	15	19	2	17	64798.63	-0.036				

20	9	12	19	8	12	62696.91	-0.088	61636.89	0.02	60648.50	0.06
20	9	11	19	8	11	62696.91	-0.083	61636.89	0.03	60648.50	0.07
20	10	11	19	9	11	66395.04	0.071			64187.75	-0.08
20	10	10	19	9	10	66395.04	0.072			64187.75	-0.08
20	11	10	19	10	10	70087.02	-0.029	68860.60	0.08	67722.17	-0.02
20	11	9	19	10	9	70087.02	-0.029	68860.60	0.08	67722.17	-0.02
20	12	9	19	11	9	73776.04	0.064	72466.68	-0.03		
20	12	8	19	11	8	73776.04	0.064	72466.68	-0.03		
20	17	3	20	16	5	60791.60	0.100				
20	17	4	20	16	4	60791.60	0.100				
20	18	2	20	17	4	64479.29	-0.032				
20	18	3	20	17	3	64479.29	-0.032				
21	4	17	20	1	19	64389.24	0.030				
21	5	16	20	2	18	66353.84	0.040				
21	8	14	20	7	14	60540.53	-0.266				
21	8	13	20	7	13	60540.53	0.129				
21	9	13	20	8	13	64257.28	0.025	63179.65	-0.09	62174.13	0.08
21	9	12	20	8	12	64257.28	0.036	63179.65	-0.09	62174.13	0.09
21	11	11	20	10	11	71653.27	-0.129	70408.92	-0.10	69253.07	0.05
21	11	10	20	10	10	71653.27	-0.129	70408.92	-0.10	69253.07	0.05
21	17	4	21	16	6	60788.11	0.026				
21	17	5	21	16	5	60788.11	0.026				
21	18	3	21	17	5	64476.36	-0.071				
21	18	4	21	17	4	64476.36	-0.071				
22	5	17	21	2	19	68104.00	-0.098				
22	8	15	21	7	15	62090.32	-0.347	61081.13	-0.25	60135.33	-0.23
22	8	14	21	7	14	62090.32	0.433	61081.13	0.38	60135.33	0.28
22	9	14	21	8	14	65815.32	0.018	64720.58	0.01		
22	9	13	21	8	13	65815.32	0.042	64720.58	0.03		
22	10	13	21	9	13	69521.71	-0.026				
22	10	12	21	9	12	69521.71	-0.025				
22	11	12	21	10	12	73218.66	-0.120	71956.52	-0.11		
22	11	11	21	10	11	73218.66	-0.120	71956.52	-0.11		
22	17	5	22	16	7	60784.21	0.041				
22	17	6	22	16	6	60784.21	0.041				
22	18	4	22	17	6	64473.12	-0.002				
22	18	5	22	17	5	64473.12	-0.002				
23	5	18	22	2	20	70076.50	-0.032				
23	7	17	22	6	17	59864.95	-0.048				
23	7	16	22	6	16	59835.41	0.048				
23	8	16	22	7	16	63636.50	-0.085	62610.91	-0.08	61648.79	-0.10
23	8	15	22	7	15	63635.17	0.073	62609.76	-0.02	61647.96	0.05
23	9	15	22	8	15	67370.74	-0.095	66259.03	-0.04		
23	9	14	22	8	14	67370.74	-0.043	66259.03	0.00		
23	10	14	22	9	14	71082.73	-0.031				
23	10	13	22	9	13	71082.73	-0.030				
23	17	6	23	16	8	60779.64	-0.068				
23	17	7	23	16	7	60779.64	-0.068				
23	18	5	23	17	7	64469.31	-0.044				
23	18	6	23	17	6	64469.31	-0.044				
24	7	18	23	6	18	61391.71	-0.055				
24	7	17	23	6	17	61342.61	0.101				
24	8	17	23	7	17	65178.16	0.057	64136.53	0.02		

24	8	16	23	7	16	65175.53	0.172	64134.36	0.08
24	9	16	23	8	16	68923.40	-0.126	67794.82	-0.13
24	9	15	23	8	15	68923.40	-0.020	67794.82	-0.04
24	10	15	23	9	15	72641.91	-0.018		
24	10	14	23	9	14	72641.91	-0.015		
24	17	7	24	16	9	60774.71	0.057		
24	17	8	24	16	8	60774.71	0.057		
24	18	6	24	17	8	64465.06	-0.028		
24	18	7	24	17	7	64465.06	-0.028		
25	7	19	24	6	19	62914.05	0.027		
25	7	18	24	6	18	62834.27	-0.046		
25	8	18	24	7	18	66714.67	-0.115	65657.49	-0.04
25	8	17	24	7	17	66709.95	0.083	65653.59	0.05
25	9	17	24	8	17	70472.93	-0.097		
25	9	16	24	8	16	70472.93	0.112		
25	10	16	24	9	16	74199.02	0.018		
25	10	15	24	9	15	74199.02	0.024		
25	17	8	25	16	10	60768.94	-0.012		
25	17	9	25	16	9	60768.94	-0.012		
25	18	7	25	17	9	64460.26	-0.020		
25	18	8	25	17	8	64460.26	-0.020		
26	6	21	25	5	21	60801.45	0.075		
26	6	20	25	5	20	59701.96	-0.030		
26	7	20	25	6	20	64432.71	0.075		
26	7	19	25	6	19	64306.95	0.094		
26	8	19	25	7	19	68246.25	0.024		
26	8	18	25	7	18	68237.71	0.058	67166.67	-0.03
26	9	18	25	8	18	72018.77	-0.196		
26	9	17	25	8	17	72018.77	0.205		
26	18	8	26	17	10	64454.91	0.025		
26	18	9	26	17	9	64454.91	0.025		
27	3	25	26	2	25	61160.13	0.014		
27	4	24	26	3	24	59939.50	-0.015		
27	5	23	26	4	23	60124.28	0.097		
27	6	22	26	5	22	62398.85	0.001		
27	6	21	26	5	21	60908.14	0.033		
27	7	21	26	6	21	65949.04	0.059		
27	7	20	26	6	20	65755.25	0.031		
27	8	20	26	7	20	69772.09	0.009		
27	8	19	26	7	19	69757.64	0.129		
27	9	19	26	8	19	73560.84	-0.110		
27	9	18	26	8	18	73560.27	0.067		
27	18	9	27	17	11	64448.94	0.084		
27	18	10	27	17	10	64448.94	0.084		
28	3	25	27	2	25	60376.15	0.069		
28	3	26	27	2	26	63520.09	-0.082		
28	4	25	27	3	25	62176.99	0.065		
28	5	24	27	4	24	62051.23	0.072		
28	6	23	27	5	23	64023.42	-0.112		
28	6	22	27	5	22	62059.01	0.114		
28	7	22	27	6	22	67465.05	-0.006		
28	7	21	27	6	21	67173.44	0.016		
28	8	21	27	7	21	71292.06	-0.054		

28	8	20	27	7	20	71267.96	0.021
29	3	26	28	2	26	63013.64	-0.094
29	4	26	28	3	26	64446.80	0.138
29	5	25	28	4	25	64031.58	-0.128
29	6	24	28	5	24	65681.06	-0.060
29	6	23	28	5	23	63164.84	-0.086
29	7	22	28	6	22	68554.42	-0.060
29	8	22	28	7	22	72806.22	-0.030
30	5	26	29	4	26	66065.29	-0.008
30	6	24	29	5	24	64244.61	-0.075
31	5	27	30	4	27	68150.23	-0.060
31	6	26	30	5	26	69116.55	0.068
31	6	25	30	5	25	65323.85	0.087
31	7	24	30	6	24	71174.29	-0.078

Table S2. Measured transition frequencies for PTS (MHz).

J″	*K*a″	*K*c″	*F″*	*J′*	*K*a′	*K*c′	*F′*	*ν*	Δ*ν*
1	1	0	1	0	0	0	1	3197.851	-0.005
1	1	0	2	0	0	0	1	3197.473	-0.001
1	1	0	0	0	0	0	1	3196.904	0.003
2	1	1	2	1	0	1	1	4324.263	-0.005
2	1	1	2	1	0	1	2	4323.476	-0.005
2	1	1	3	1	0	1	2	4323.913	-0.003
2	1	1	1	1	0	1	0	4322.975	0.000
3	0	3	2	2	0	2	1	3224.251	-0.018
3	0	3	4	2	0	2	3	3224.461	0.029
3	1	2	2	2	0	2	1	5475.620	-0.022
3	1	2	3	2	0	2	2	5476.036	-0.014
3	1	2	4	2	0	2	3	5475.874	-0.003
3	1	2	3	2	1	1	2	3302.894	-0.002
3	1	2	4	2	1	1	3	3303.129	-0.002
3	1	2	2	2	0	2	2	5476.955	-0.001
3	1	3	4	2	1	2	3	3151.943	0.003
3	1	3	3	2	1	2	2	3151.710	0.004
3	2	1	3	2	2	0	2	3231.009	0.001
3	2	1	4	2	2	0	3	3231.856	0.002
3	2	2	4	2	2	1	3	3228.224	0.004
3	2	2	3	2	2	1	2	3227.380	0.005
4	0	4	5	3	0	3	4	4294.973	-0.020
4	1	3	4	3	0	3	3	6654.560	-0.016
4	1	3	3	3	0	3	2	6654.358	-0.010
4	1	3	3	3	0	3	3	6655.546	-0.004
4	1	4	4	3	1	3	3	4201.392	0.000
4	1	3	4	3	1	2	3	4402.928	0.002
4	1	3	4	3	0	3	4	6653.703	0.002
4	1	3	5	3	0	3	4	6654.483	0.007
4	1	3	5	3	1	2	4	4403.056	0.025
4	1	4	5	3	1	3	4	4201.521	0.026
4	2	3	5	3	2	2	4	4303.428	-0.002
4	2	2	5	3	2	1	4	4312.499	0.000
4	2	3	4	3	2	2	3	4303.073	0.001
4	2	2	4	3	2	1	3	4312.142	0.002

4	2	3	3	3	2	2	2	4303.526	0.004
4	2	2	3	3	2	1	2	4312.597	0.005
5	0	5	6	4	0	4	5	5361.991	0.012
5	1	5	6	4	1	4	5	5250.192	-0.019
5	1	4	6	4	1	3	5	5501.978	-0.005
5	1	4	4	4	0	4	3	7861.400	-0.005
5	1	4	5	4	0	4	4	7861.547	0.019
5	2	4	5	4	2	3	4	5377.898	0.000
5	2	3	5	4	2	2	4	5395.980	0.000
5	2	4	6	4	2	3	5	5378.090	0.005
5	2	3	6	4	2	2	5	5396.179	0.010
6	0	6	7	5	0	5	6	6424.588	0.002
6	1	5	7	5	1	4	6	6599.690	-0.008
6	2	4	6	5	2	3	5	6483.352	-0.011
6	2	5	6	5	2	4	5	6451.881	-0.009
6	2	5	7	5	2	4	6	6452.011	0.010
6	2	4	7	5	2	3	6	6483.490	0.014
7	0	7	8	6	0	6	7	7482.124	-0.006
7	1	7	8	6	1	6	7	7344.377	-0.025
7	1	6	8	6	1	5	7	7695.875	0.011
12	12	1		11	11	1		61126.66	-0.076
12	12	0		11	11	0		61126.66	-0.076
13	12	2		12	11	2		62203.08	0.108
13	12	1		12	11	1		62203.08	0.108
13	13	1		12	12	1		66395.24	0.131
13	13	0		12	12	0		66395.24	0.131
14	12	3		13	11	3		63279.18	-0.008
14	12	2		13	11	2		63279.18	-0.008
14	13	2		13	12	2		67471.37	0.037
14	13	1		13	12	1		67471.37	0.037
14	14	1		13	13	1		71663.43	-0.002
14	14	0		13	13	0		71663.43	-0.002
15	11	5		14	10	5		60163.06	-0.055
15	11	4		14	10	4		60163.06	-0.055
15	12	4		14	11	4		64355.34	-0.032
15	12	3		14	11	3		64355.34	-0.032
15	13	3		14	12	3		68547.60	0.061
15	13	2		14	12	2		68547.60	0.061
15	14	2		14	13	2		72739.55	-0.094
15	14	1		14	13	1		72739.55	-0.094
16	11	6		15	10	6		61239.15	-0.041
16	11	5		15	10	5		61239.15	-0.041
16	13	4		15	12	4		69623.65	-0.066
16	13	3		15	12	3		69623.65	-0.066
16	14	3		15	13	3		73815.78	-0.057
16	14	2		15	13	2		73815.78	-0.057
17	11	7		16	10	7		62315.24	0.055
17	11	6		16	10	6		62315.24	0.055
18	11	8		17	10	8		63391.12	0.042
18	11	7		17	10	7		63391.12	0.042
19	10	10		18	9	10		60273.80	0.146
19	10	9		18	9	9		60273.80	0.146
20	10	11		19	9	11		61348.94	0.000

20	10	10	19	9	10	61348.94	0.000
21	10	12	20	9	12	62423.91	-0.074
21	10	11	20	9	11	62423.91	-0.074
22	10	13	21	9	13	63498.78	0.031
22	10	12	21	9	12	63498.78	0.031
23	9	15	22	8	15	60375.99	0.070
23	9	14	22	8	14	60375.99	0.070
23	10	14	22	9	14	64573.22	0.019
23	10	13	22	9	13	64573.22	0.019
24	9	16	23	8	16	61448.84	0.018
24	9	15	23	8	15	61448.84	0.018
24	10	15	23	9	15	65647.33	0.033
24	10	14	23	9	14	65647.33	0.033
25	9	17	24	8	17	62521.20	0.067
25	9	16	24	8	16	62521.20	0.067
25	10	16	24	9	16	66720.98	-0.018
25	10	15	24	9	15	66720.98	-0.018
26	9	18	25	8	18	63592.72	-0.068
26	9	17	25	8	17	63592.72	-0.068
26	10	17	25	9	17	67794.22	-0.037
26	10	16	25	9	16	67794.22	-0.037
27	8	20	26	7	20	60451.70	-0.006
27	8	19	26	7	19	60451.70	0.031
27	9	19	26	8	19	64663.69	-0.031
27	9	18	26	8	18	64663.69	-0.030
28	8	21	27	7	21	61518.16	-0.044
28	8	20	27	7	20	61518.16	0.019
28	9	20	27	8	20	65733.78	-0.079
28	9	19	27	8	19	65733.78	-0.078
29	8	22	28	7	22	62583.24	-0.114
29	8	21	28	7	21	62583.24	-0.009
31	7	25	30	6	25	60448.75	-0.047
31	7	24	30	6	24	60441.83	0.137

Table S3. Measured transition frequencies for OTS(MHz).

J''	K_a''	K_c''	F''	J'	K_a'	K_c'	F'	ν	$\Delta\nu$
1	1	0	0	0	0	0	1	2581.311	-0.006
1	1	0	2	0	0	0	1	2582.010	-0.021
1	1	0	1	0	0	0	1	2582.501	-0.007
2	0	2	2	1	0	1	2	2901.407	0.004
2	0	2	1	1	0	1	0	2901.548	0.004
2	0	2	3	1	0	1	2	2902.072	-0.009
2	0	2	1	1	0	1	1	2903.056	-0.011
2	1	2	2	1	1	1	1	2737.889	0.005
2	1	2	3	1	1	1	2	2738.521	-0.006
2	1	2	1	1	1	1	1	2738.678	0.000
2	1	2	1	1	1	1	0	2738.988	-0.022
2	1	1	2	1	1	0	1	3117.719	0.008
2	1	1	1	1	1	0	1	3117.948	0.016
2	1	1	3	1	1	0	2	3118.337	0.007
2	1	1	1	1	1	0	0	3119.122	-0.001
2	1	1	3	1	0	1	2	4236.109	0.002

2	1	1	2	1	0	1	1	4236.585	0.012
2	1	1	1	1	0	1	1	4236.799	0.004
3	0	3	3	2	0	2	3	4289.797	-0.032
3	0	3	4	2	0	2	3	4290.539	-0.030
3	0	3	3	2	0	2	2	4290.539	0.032
3	0	3	2	2	0	2	2	4291.509	0.002
3	1	3	3	2	1	2	3	4091.516	-0.003
3	1	3	3	2	1	2	2	4092.018	-0.010
3	1	3	4	2	1	2	3	4092.229	0.005
3	1	3	2	2	1	2	2	4092.968	-0.014
3	1	2	3	2	1	1	2	4659.478	0.006
3	1	2	4	2	1	1	3	4659.650	-0.006
3	1	2	2	2	0	2	1	5993.420	0.002
3	1	2	4	2	0	2	3	5993.672	-0.009
3	1	2	3	2	0	2	2	5994.050	0.016
3	2	2	3	2	2	1	2	4391.961	0.013
3	2	2	4	2	2	1	3	4392.617	0.016
3	2	2	2	2	2	1	1	4392.979	0.016
3	2	1	3	2	2	0	2	4493.882	-0.015
3	2	1	4	2	2	0	3	4494.521	0.010
3	2	1	2	2	2	0	1	4494.881	0.015
4	0	4	4	3	0	3	4	5618.674	0.005
4	0	4	5	3	0	3	4	5619.422	-0.047
4	0	4	4	3	0	3	3	5619.422	0.013
4	0	4	3	3	0	3	2	5619.422	0.007
4	0	4	3	3	0	3	3	5620.415	0.000
4	1	4	5	3	1	3	4	5430.464	0.004
4	1	3	5	3	1	2	4	6176.491	-0.009
4	1	3	3	3	1	2	2	6176.491	-0.003
4	1	3	3	3	0	3	2	7879.437	-0.023
4	1	3	5	3	0	3	4	7879.601	-0.010
4	1	3	4	3	0	3	3	7879.919	-0.014
4	2	3	4	3	2	2	3	5835.871	0.006
4	2	3	5	3	2	2	4	5836.171	0.023
4	2	2	4	3	2	1	3	6072.919	0.024
4	2	2	5	3	2	1	4	6073.178	0.044
4	2	2	3	3	2	1	2	6073.178	-0.024
5	0	5	6	4	0	4	5	6900.569	0.004
5	1	5	6	4	1	4	5	6752.373	0.002
5	1	4	6	4	1	3	5	7655.594	-0.007
5	2	4	5	4	2	3	4	7262.346	-0.007
5	2	4	6	4	2	3	5	7262.505	-0.004

J''	K_a''	K_c''	J'	K_a'	K_c'	ν_A (MHz)	$\Delta\nu$ (MHz)	ν_E (MHz)	$\Delta\nu$ (MHz)
18	17	2	17	16	2	60125.17	0.021	60124.550	-0.003
18	17	1	17	16	1	60125.17	0.021		
18	18	1	17	17	1	62162.32	0.070	62161.67	0.020
18	18	0	17	17	0	62162.32	0.070		
19	17	3	18	16	3	61597.68	-0.032	61597.14	0.027
19	17	2	18	16	2	61597.68	-0.032		
19	18	2	18	17	2	63635.03	0.049	63634.39	0.012
19	18	1	18	17	1	63635.03	0.049		
19	19	1	18	18	1	65672.05	-0.006	65671.52	0.071

19	19	0	18	18	0	65672.05	-0.006		
20	16	5	19	15	5	61031.70	-0.025	61031.11	-0.016
20	16	4	19	15	4	61031.70	-0.025		
20	17	4	19	16	4	63070.00	0.029	63069.39	0.022
20	17	3	19	16	3	63070.00	0.029		
20	18	3	19	17	3	65107.47	-0.082	65106.90	-0.045
20	18	2	19	17	2	65107.47	-0.082		
20	19	1	19	18	1			67145.070	-0.007
20	20	1	19	19	1	69181.72	-0.103	69181.180	-0.029
20	20	0	19	19	0	69181.72	-0.103	69182.140	0.017
21	15	7	20	14	7	60462.06	0.003	60461.41	-0.048
21	15	6	20	14	6	60462.06	0.003	60462.32	-0.052
21	16	6	20	15	6			62502.11	-0.032
21	16	5	20	15	5			62502.99	-0.065
21	17	5	20	16	5	64541.81	0.022	64541.28	0.097
21	17	4	20	16	4	64541.81	0.022		
21	18	4	20	17	4	66579.78	-0.072	66579.31	0.067
21	18	3	20	17	3	66579.78	-0.072		
21	19	2	20	18	2	68617.38	0.035	67144.30	0.137
21	19	3	20	18	3	68617.38	0.035		
22	15	8	21	14	8	61930.60	0.063	61929.99	0.055
22	15	7	21	14	7	61930.60	0.063		
22	16	7	21	15	7	63972.82	-0.074	63972.36	0.072
22	16	6	21	15	6	63972.82	-0.074		
22	16	7	21	15	7				
22	17	6	21	16	6	66012.96	-0.055	66012.33	-0.076
22	17	5	21	16	5	66012.96	-0.055		
22	20	3	21	19	3	72127.11	0.018	72126.46	-0.012
22	20	2	21	19	2	72127.11	0.018		
23	14	10	22	13	10	61348.33	0.072	61347.72	0.065
23	14	9	22	13	9	61348.33	0.072		
23	15	9	22	14	9			63396.93	0.048
23	15	8	22	14	8			63397.77	-0.025
23	16	8	22	15	8	65441.95	-0.015	65441.38	0.024
23	16	7	22	15	7	65441.95	-0.015	65442.24	-0.028
24	13	12	23	12	12	60746.25	-0.035	60745.66	-0.016
24	13	11	23	12	11	60746.25	-0.029	60746.56	-0.039
24	14	11	23	13	11			62808.79	-0.009
24	14	10	23	13	10			62809.61	-0.106
25	12	14	24	11	14	60105.65	0.026	60104.79	-0.031
25	13	13	24	12	13	62197.15	0.119	62196.46	0.049
25	13	12	24	12	12	62197.15	0.138		
25	14	12	24	13	12			64267.00	0.064
25	14	11	24	13	11			64267.84	-0.013
26	12	15	25	11	15			61536.35	-0.047
26	12	14	25	11	14			61537.20	-0.078
26	13	14	25	12	14	63642.76	-0.037	63642.12	-0.037
26	13	13	25	12	13	63642.76	0.015		
27	11	17	26	10	16	60755.46	-0.060	60752.31	0.074
27	11	16	26	10	16	60761.70	0.054	60763.71	0.077
27	11	17	26	10	17	60790.96	0.027	60788.71	0.033
27	11	16	26	10	17	60797.03	-0.029	60800.18	0.106
27	12	15	26	11	15	62960.02	0.008		

27	12	16	26	11	16	62960.91	-0.097	62959.41	-0.008
28	11	18	27	10	17	62109.03	-0.063	62107.19	-0.010
28	11	17	27	10	17	62122.42	-0.074	62123.62	-0.014
28	11	18	27	10	18	62181.63	0.010	62180.21	-0.002
29	11	18	28	10	18	63449.59	0.030	63450.11	0.031
29	11	19	28	10	19	63564.05	-0.025	63563.24	-0.052

Table S4. Measured transition frequencies for SUA (MHz).

J″	K_a″	K_c″	I″	F″	*J*′	K_a′	K_c′	I′	F′	*ν*	Δ*ν*
3	0	3			2	0	2			3231.655	-0.010
4	1	4	2	6	3	1	3	2	5	4209.013	-0.018
4	1	4	2	5	3	1	3	2	4	4209.137	-0.015
4	1	3	2	6	3	1	2	2	5	4415.203	0.067
4	1	3	2	5	3	1	2	2	4	4415.203	-0.054
4	2	3	2	6	3	2	2	2	5	4313.251	-0.003
5	0	5			4	0	4			5373.400	0.038
5	1	5			4	1	4			5259.538	-0.019
5	1	4			4	1	3			5517.064	0.027
5	2	4	2	6	4	2	3	2	5	5390.564	-0.007
5	2	4	2	7	4	2	3	2	6	5390.35	-0.015
5	2	4	1	6	4	2	3	1	5	5390.176	-0.002
5	2	3	2	7	4	2	2	2	6	5409.51	-0.001
6	0	6		8	5	0	5	2	7	6437.683	0.001
6	1	6		8	5	1	5	2	7	6309.023	0.041
6	1	5		8	5	1	4	2	7	6617.587	-0.026
6	2	5	1	7	5	2	4	1	6	6466.541	-0.015
6	2	5	2	8	5	2	4	2	7	6466.668	0.001
6	2	5	2	7	5	2	4	2	6	6466.805	0.014
6	2	4	2	8	5	2	3	2	7	6499.996	0.014
7	0	7			6	0	6			7496.667	0.005
7	1	7			6	1	6			7357.132	-0.017
7	1	6			6	1	5			7716.524	-0.015
7	2	6			6	2	5			7542.016	0.009
7	2	5			6	2	4			7594.828	0.009
7	3	5	2	9	6	3	4	2	8	7556.798	0.009
7	3	4	2	9	6	3	3	2	8	7558.195	-0.005
7	3	4	1	8	6	3	3	1	7	7558.044	-0.005
7	3	4	2	8	6	3	3	2	7	7558.355	-0.011

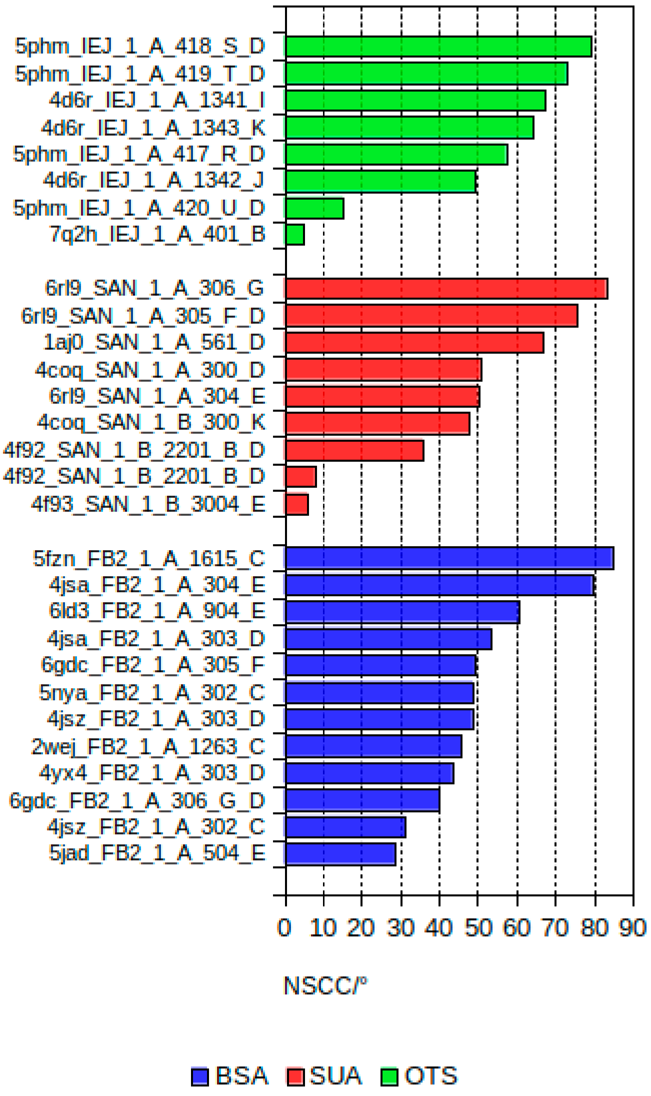

Figure S1. ∠CCSN torsion angle values found in PDB for BSA, SUA and OTS.
